# Comparative analysis of the ten Tusscher and Tomek human ventricular cell models at cellular, tissue, and organ levels: Implications for post‐infarct ventricular tachycardia simulation

**DOI:** 10.14814/phy2.70435

**Published:** 2025-07-10

**Authors:** Ruiqing Dong, Zhenyin Fu, Chuxin Zhang, Yumeng Liu, Yiming Wang, Nan Zhang, Zefeng Wang, Jun Hou, Ling Xia, Yongquan Wu, Shijie Zhou, Dongdong Deng

**Affiliations:** ^1^ Department of Cardiology The Fourth Affiliated Hospital of Soochow University Suzhou China; ^2^ College of Biomedical Engineering & Instrument Science Zhejiang University Hangzhou Zhejiang China; ^3^ School of Biomedical Engineering Dalian University of Technology Dalian Liaoning China; ^4^ Department of Radiology The First Affiliated Hospital of Soochow University Suzhou China; ^5^ Department of Radiology Beijing Anzhen Hospital, Capital Medical University Beijing China; ^6^ Department of Cardiology Beijing Anzhen Hospital, Capital Medical University Beijing China; ^7^ Department of Biomedical Engineering Worcester Polytechnic Institute Worcester Massachusetts USA

**Keywords:** actional potential duration, conduction velocity, scar‐related tissue, ten Tusscher model, Tomek model

## Abstract

Computational modeling is a tool for exploring both normal electrical propagation in healthy hearts and cardiac arrhythmias in patients. While numerous human ventricular cell models exist, the ten Tusscher (TT2) model is one of the most used for simulating ventricular arrhythmia. Recently, the Tomek model has been proposed, offering improved accuracy by better reproducing key depolarization, repolarization, and calcium dynamics in healthy ventricular cardiomyocytes. However, a quantitatively comprehensive comparison of these models at the single‐cell, tissue, and organ levels has not been conducted. This study systematically compared the TT2 and Tomek models by evaluating electrophysiological parameters and reentrant properties in 0‐dimensional(0D), 1‐dimensional(1D), 2‐dimensional (2D), and 3‐dimensional (3D) simulations. Additionally, the effects of ion currents modifications to simulate ischemic scar‐related tissue were analyzed. These results reveal that although the TT2 and Tomek models demonstrate distinct 0D, 1D, and 2D characteristics, their 3D reentrant properties—specifically in terms of reentry locations and critical conduction channels—are highly comparable. Therefore, both models are suitable for simulating post‐infarct ventricular tachycardia (VT), as their shared 3D features effectively capture the essential mechanisms underlying this arrhythmia.

## 1 INTRODUCTION

Computational cardiac modeling has become a powerful tool for exploring the mechanisms underlying normal and pathological cardiac electrophysiology (Bishop et al., 2010; Gong et al., 2007; Trayanova et al., 2024; Vigmond et al., 2009; Zhao et al., 2012). It has been employed in interpreting experimental and clinical data (Campos et al., 2022; Gong et al., 2007; Krueger et al., 2012; Nánási et al., 2021; Vigmond et al., 2009), offering insights into arrhythmogenesis (Campos et al., 2019; Zahid et al., 2016; Zhao et al., 2012) and therapeutic interventions (Doste et al., 2020; Moreno et al., 2011; Zhou et al., 2021). Notably, personalized cardiac models have demonstrated significant promise in investigating cardiac arrhythmias and optimizing individualized treatment strategies (Boyle et al., 2019; Prakosa et al., 2018).

One of the critical determinants of a model's accuracy lies in the selection of the single‐cell action potential model, which serves as the foundation for multiscale simulations. Among the many human ventricular cell models available (Asakura et al., 2014; Carro et al., 2011; Grandi et al., 2009; O'Hara et al., 2011; Tomek et al., 2019), the ten Tusscher (TT2) model (ten Tusscher et al., 2004; ten Tusscher & Panfilov, 2006) is widely adopted for its balance between computational efficiency and physiological relevance (O'Hara et al., 2021; Prakosa et al., 2018; Shade et al., 2021; Tong et al., 2021). Despite its widespread use, the TT2 model relies on experimental data that are now somewhat dated, limiting its capacity to capture the latest advances in clinical cardiac electrophysiology.

The recently developed Tomek model addresses critical limitations of earlier formulations by incorporating state‐of‐the‐art experimental data on ion channel kinetics and calcium dynamics (Tomek et al., 2019). This model enhances the physiological accuracy of simulations, particularly for human ventricular cardiomyocytes, and is uniquely positioned to provide deeper mechanistic insights into arrhythmia pathways. Despite its potential advantages, no systematic comparison has been made to assess the relative strengths and weaknesses of both the TT2 and Tomek models across cellular, tissue, and organ levels.

This study aims to bridge this gap by performing a comprehensive evaluation of the TT2 and Tomek models. Key electrophysiological properties, including action potential morphology, ion currents, action potential duration (APD) restitution, conduction velocity (CV) restitution, and reentrant behaviors, were compared in 0D, 1D, 2D, and 3D simulations. In addition, we explored their applicability in representing the electrophysiological characteristics of scar‐related tissue found in ischemic heart conditions as depicted in late gadolinium enhancement (LGE) images. By analyzing these factors, this study aims to guide researchers in selecting the most appropriate model for specific applications and inspire the development of next‐generation computational models for cardiac arrhythmia research.

## 2 METHODS

### 2.1 Electrophysiological modeling at the cellular scale

This study employed two established human ventricular models: the TT2 (ten Tusscher et al., 2004; ten Tusscher & Panfilov, 2006) and the Tomek (Tomek et al., 2019) models, integrated into the open‐source software openCARP (Plank et al., 2021) (https://opencarp.org/). Modifications to these models, based on experimental data, simulated the electrophysiological behavior of myocardial tissue in the gray zone (Arevalo et al., 2013; Tong et al., 2021). Reductions were applied to the original values of peak sodium current (INa), L‐type calcium current (ICaL), rapid delayed rectifier current (IKr), and slow delayed rectifier current (IKs), setting them to 38%, 31%, 30%, and 20%, respectively (Arevalo et al., 2013; Tong et al., 2021).

In the study, the 0D single‐cell models were used to obtain action potentials and important transmembrane ion currents, which were then discussed and analyzed. 0D single‐cell simulations (Cherry et al., 2008) were used to characterize action potential duration (APD) restitution across dynamic and S1‐S2 protocols. For dynamic APD restitution, 20 sets of pre‐stimulations were applied, with each set consisting of 10 pulses at a pulse width of 1 ms. The cycle interval (CI) for the first set was 1000 ms and was systematically reduced by 20 ms after each subsequent pacing set, reaching a minimum of 50 ms. For S1‐S2 APD restitution, the protocol began with a pre‐pacing phase of 20 stimuli at a basic cycle length (BCL) of 1000 ms, followed by a train of 10 S1 stimuli. Afterward, the CI of S2 stimuli was reduced incrementally by 20 ms, starting at 1000 ms and decreasing to a minimum of 50 ms.

The 1D cable model (Cherry et al., 2008), using a 10 mm linear cable model discretized into finite elements with an average edge length of 0.1 mm to determine the conduction velocity (CV) restitution curve. Pacing was applied to the left side of the 10 mm linear cable with 100 pre‐stimulations at a BCL of 600 ms. Subsequently, the CI for the S2 stimulus was systematically reduced from an initial 1000 ms in increments of 25 ms, reaching a final CI of 275 ms. Activation times for each S2 beat were recorded. The CV along the cable was calculated by measuring the activation time at two points—2.5 mm and 7.5 mm from the pacing site—and dividing the time by the 5 mm distance between these points. The cable model's electrical properties were set with an intracellular longitudinal tissue conductivity of 0.174 S/m and an extracellular longitudinal tissue conductivity of 0.625 S/m.

### 2.2 Modeling tissue‐scale electrophysiological properties

For 2D tissue‐scale electrophysiological dynamics, a specialized ultra‐thin 3D model (4 cm × 4 cm × 0.04 cm) was developed using adaptive tetrahedral meshing integrating TT2 and Tomek ventricular cell models to compare their electrophysiological behavior. With an average mesh resolution of 116.5 ± 28.6 μm, the model approximated planar dynamics by minimizing through‐thickness variability, effectively simulating 2D electrical interactions in a sheet‐like architecture. The isotropic conductivity was standardized at 0.008 S/m across the tissue to simulate electrical propagation. A cross‐field stimulation protocol was used to induce reentry phenomena. The first stimulus, at 60 μA/cm^2^, was applied to a narrow section on the left side of the tissue model, followed by a second stimulus of equal intensity to the lower half of the model after a controlled delay. The timing of the second stimulus was adjusted based on CV differences: for normal tissues, a delay of 360 ms was used, while for gray zones, the delay was extended to 550 ms to account for slower CVs caused by pathological changes.

To ensure stable reentry within the simulation grid, the timing of the second stimulus was further refined based on observed conduction speeds. Cellular states were initialized from precomputed single‐cell simulations involving 100 pacing beats at a BCL of 600 ms, with final states captured for use in the tissue‐scale simulations.

Additionally, to address potential boundary effects observed during initial simulations with the Tomek model, the tissue model dimensions were expanded to 8 cm × 4 cm × 0.04 cm. The updated mesh had an average resolution of 231.905 ± 102.736 μm. The adjustment ensured the model's capacity to sustain prolonged phenomena while preserving the original electrophysiological parameters.

### 2.3 Three‐dimensional human ventricle simulation

A patient‐specific human ventricular anatomic model was used to evaluate the efficacy of the TT2 and Tomek single‐cell models in simulating 3D human heart electrophysiology. Details of this model have been described previously (Tong et al., 2021). In this study, a significant modification was the inclusion of programmed electrical stimulation to assess the models' ability to replicate electrical activities and reentrant arrhythmias. In the TT2 model, electrical stimulations were applied at 19 different cardiac sites, selected according to the American Heart Association Classification Standards (Cerqueira et al., 2002). These sites included 17 locations within the left ventricle, one in the right ventricular outflow tract, and one in the inferior region of the right ventricle. In the Tomek model, due to computational resource constraints, we limited our Tomek model simulations to the sites that had already been shown to induce reentry in the TT2 model. We then analyzed the reentry induction and various morphological parameters for those sites in the Tomek model.

The stimulation protocol involved an initial pacing (S1) at each site, delivered at a cycle length of 600 ms for six consecutive cycles. Following this, a pre‐stimulus (S2) was applied immediately after the last S1 pulse, with an initial interval of 250 ms. If the S2 fails to induce a reentry, the interval was reduced by 10 ms increments. Additional stimuli (S3 and S4) were applied if needed, using the same approach. If reentry ventricular tachycardia (VT) could not be triggered after the S4 stimulus, the site was considered incapable of initiating reentry under the given conditions.

### 2.4 Simulation protocols

The propagation of electrical activity in 2D and 3D heart models was simulated by solving a monodomain reaction–diffusion partial differential equation, utilizing the finite‐element method (Plank et al., 2008). These simulations were conducted in the openCARP simulation environment (Plank et al., 2021). The computational work was performed using high‐performance computing resources at Dalian University of Technology, China, ensuring that the simulations benefited from substantial processing capabilities.

To accurately capture electrical activity dynamics, the Rush‐Larsen method (Rush & Larsen, 1978) was used to update gating variables for time‐dependent ionic currents. This approach was selected for its computational stability and efficiency, specifically in addressing the fast kinetics inherent to cardiac cells. For other currents or/and ion concentrations, the Forward Euler method (Korhonen & Tavi, 2008) was applied, leveraging its simplicity and suitability for iterative calculations in cardiac electrophysiology simulations.

To compare the electrophysiological parameters between the TT2 and Tomek models, we initially integrated simulations using 0D single‐cell simulations, 1D cable simulations, 2D sheet‐like models, and 3D ventricle models with a 10‐microsecond time step to accurately replicate physiological conditions.

We meticulously controlled the temporal resolution of these simulations to capture the changes in electrophysiological parameters. Specifically, we further evaluated time steps ranging from 5 to 30 microseconds for each individual model. This means the TT2 model was tested across this range of time steps, and separately, the Tomek model was also tested across the same range. This comprehensive evaluation was performed across multiple scales, including 0D single‐cell simulations, 1D cable simulations, 2D sheet‐like models, and 3D ventricle models.

## 3 RESULTS

### 3.1 Electrophysiological characterization of the Tomek and TT2 models at the cellular level

We assessed the single‐cell electrophysiological properties of the Tomek and TT2 models in endocardial (ENDO) tissue. Results for epicardial (EPI) and middle (MCELL) tissue layers are provided in the Appendix S1.

#### 3.1.1 Action potential, APD, and CV restitution

**FIGURE 1:**
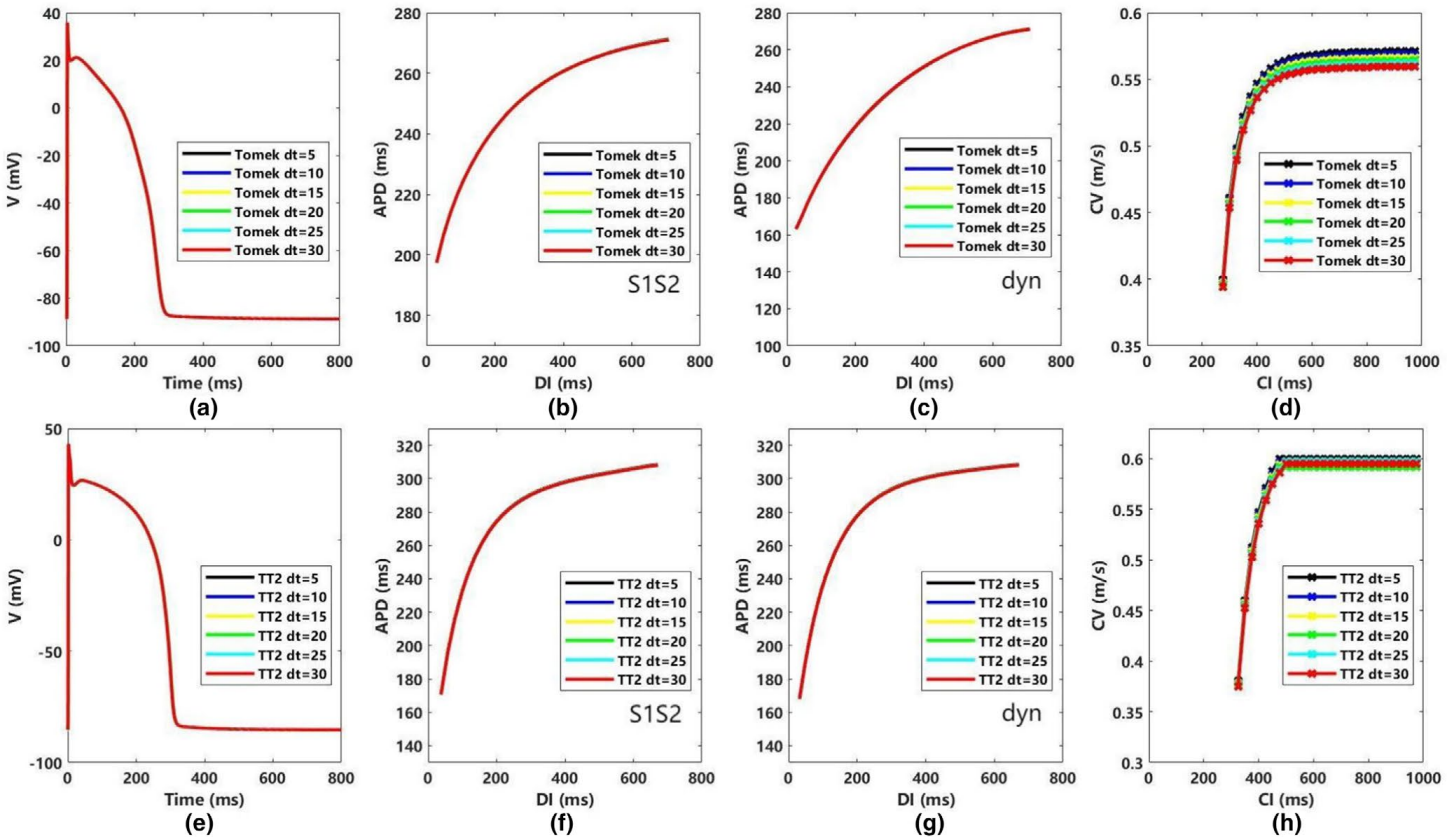
The figure compares the membrane potential, APD restitution curves, and CV restitution curves for the TT2 and Tomek models (ENDO tissue) across time steps (5–30 μs). (a) The membrane potential with different timesteps; (b) APD restitution curves in the S1‐S2 protocol with different timesteps; (c) APD restitution curves in the dynamic protocol with different timesteps; (d) CV restitution curves with different timesteps. (e) The membrane potential with different timesteps; (f) APD restitution curves in the S1‐S2 protocol with different timesteps; (g) APD restitution curves with the dynamic protocol with different timesteps; (h) CV restitution curves with different timesteps; APD represents APD90; CI, coupling interval; CV, conduction velocity; DI, diastolic interval.

Figure 1a–d shows the action potential morphology and APD restitution curves (using both the dynamic and S1–S2 protocols) for the Tomek model at various time steps (5–30 μs). The morphology and APD restitution curves in the Tomek model remain nearly identical across time steps, while the CV restitution curve (Figure 1d) shows minor variations. When the coupling interval (CI) is 900 ms, CV values range from 0.571 m/s at a 5 μs time step to 0.5596 m/s at a 30 μs time step, indicating a slight decrease in CV as the time step increases.

A similar pattern is observed for the TT2 model (Figure 1e–h), though its CV is less sensitivity to changes in time step. At a CI of 900 ms, CV values range from 0.5999 m/s at 5 μs to 0.5949 m/s at 30 μs (Figure 1h). Unlike the Tomek model, the TT2 model does not display a strictly linear relationship between CV and time step and generally has a faster CV than the Tomek model.

#### 3.1.2 Ion currents and action potential morphology

**FIGURE 2:**
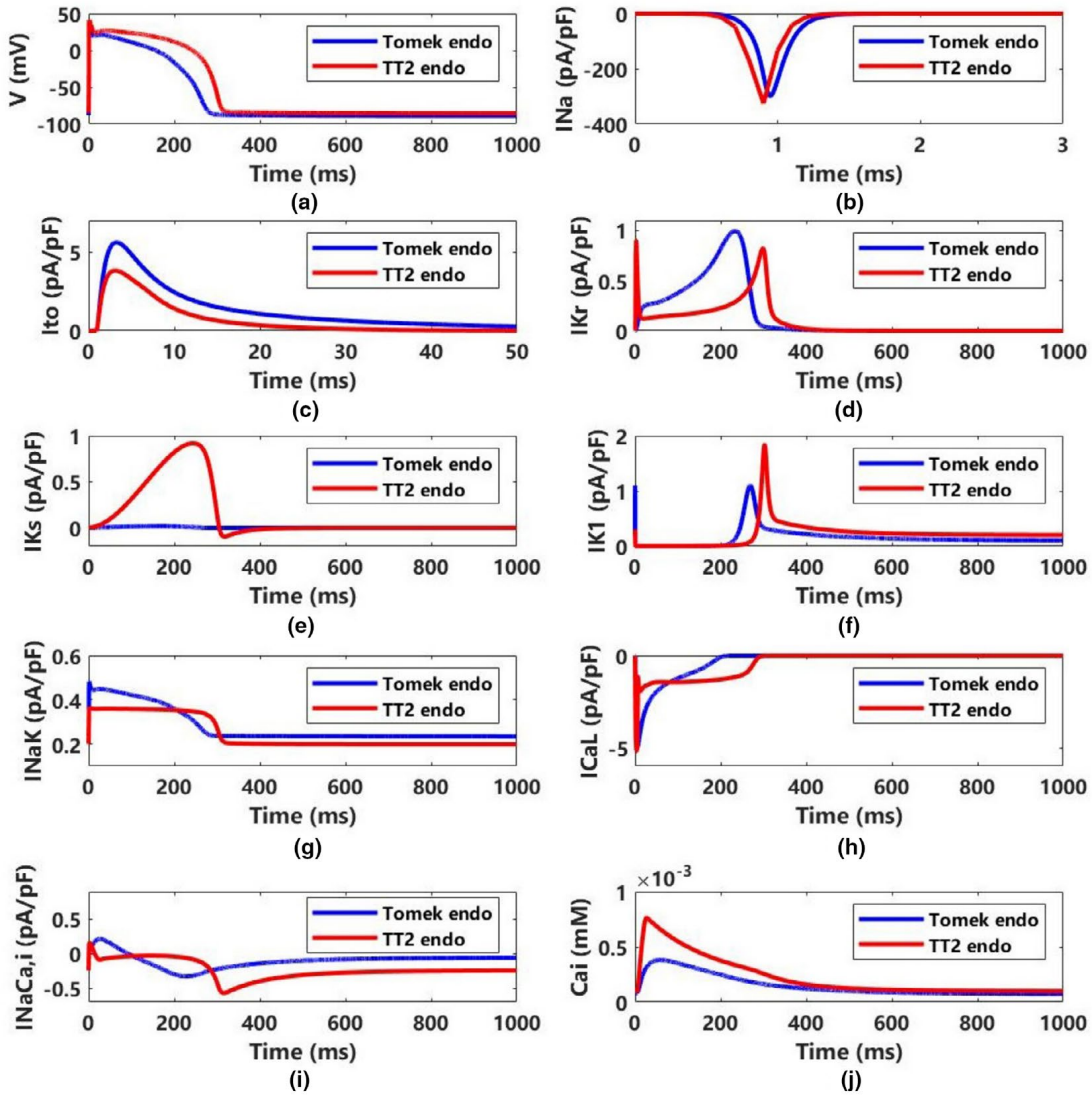
The morphologies of the action potential and major ion currents in the Tomek and the TT2 models (ENDO) with 10 μs timestep. (a) The membrane potentials, (b–j) major ion currents, including I_Na_, I_to_, I_Kr_, I_Ks_, I_K1_, I_NaK_, I_CaL_, I_NaCa,i_, and [Ca^2+^]_i_. ENDO, endocardial.

Figure 2 compares action potentials and major ion currents between the Tomek and TT2 models; additional details for endocardial and middle cells are provided in Figures S1 and S2. The TT2 model has a higher peak action potential (~41.32 mV) and a longer APD (306.09 ms) than the Tomek model (~33.26 mV, 271.73 ms). The Tomek model shows a more negative resting potential (−88.77 mV vs. −85.41 mV), attributed to differences in IK1 currents.

Table 1 illustrates quantitative differences in major ion currents between the two models.

- INa (Figure 2b): The Tomek model activates INa later and with a smaller peak magnitude than the TT2 model (−295.5 pA/pF vs. −325.7 pA/pF).
- Ito (Figure 2c): The transient outward current is larger in the Tomek model (5.599 pA/pF vs. 3.795 pA/pF).
- IKr (Figure 2d): The TT2 model shows a narrow early spike of IKr with a smaller amplitude than in the Tomek model (0.8179 pA/pF vs. 0.9941 pA/pF).
- IKs (Figure 2e): The TT2 model exhibits a much larger IKs (0.9179 pA/pF vs. 0.01739 pA/pF in Tomek).
- IK1 (Figure 2f): Both models have two peaks in IK1, but the TT2 model's magnitude is larger (1.835 pA/pF vs. 1.101 pA/pF).
- INaK (Figure 2g): The TT2 model has a smaller INaK peak than Tomek (0.3625 pA/pF vs. 0.4825 pA/pF).
- ICaL (Figure 2h): TT2 exhibits a larger peak ICaL than Tomek (−5.185 pA/pF vs. −4.986 pA/pF) and decreases faster, followed by a plateau phase.
- INaCa,i (Figure 2i): The TT2 model includes only the bulk myoplasm component, whereas Tomek divides INaCa into junctional subspace and bulk myoplasm, leading to distinct waveforms.
- [Ca2+]i (Figure 2j): The TT2 model has higher intracellular calcium levels (0.7612 μM vs. 0.3764 μM in Tomek).

**TABLE 1:** Peak value of different ion currents between TT2 and Tomek models.

Model	INa (pA/pF)	I_to_ (pA/pF)	I_Kr_ (pA/pF)	I_Ks_ (pA/pF)	I_k1_ (pA/pF)	I_NaK_ (pA/pF)	I_CaL_ (pA/pF)	[Ca2+]i(μM)	INaCa,i
TT2	−325.7	3.795	0.8179	0.9179	1.835	0.3625	−5.185	0.7612	Bulk myoplasm component
Tomek	−295.5	5.599	0.9941	0.01739	1.101	0.4825	−4.986	0.3764	Junctional subspace + bulk myoplasm

#### 3.1.3 APD restitution

**FIGURE 3:**
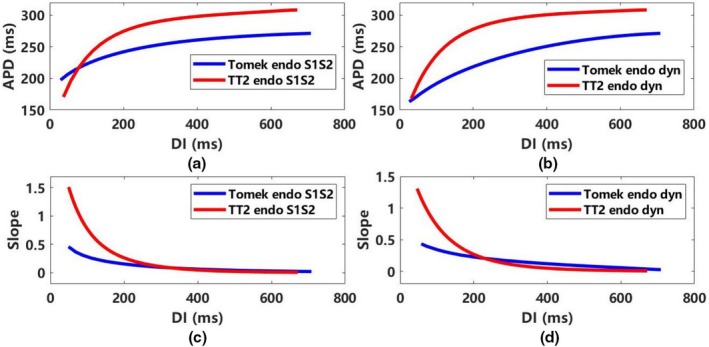
The APD restitution curves of S1‐S2 and dynamic protocol in the Tomek and the TT2 models (ENDO) with 10 μs timestep. (a) APD restitution curves with S1‐S2 protocol in the TT2 and the Tomek models, (b) APD restitution curves with dynamic protocol in the TT2 and the Tomek models, (c) the slope of APD restitution curves with S1‐S2 protocol in the TT2 and the Tomek models, (d) the slope of APD restitution curves with dynamic protocol in the TT2 and the Tomek models. APD represents APD_90_; DI, diastolic interval; dyn, dynamic protocol; ENDO, endocardial; S1S2, S1‐S2 protocol.

Figure 3 illustrates APD restitution curves under S1–S2 and dynamic protocols in the Tomek and TT2 models; additional details for endocardial and middle cells are provided in Figures S3 and S4. For the S1–S2 protocol (Figure 3a), the Tomek model's APD is shorter than TT2's when the diastolic interval (DI) is >77 ms. Under the dynamic protocol (Figure 3b), the Tomek model consistently demonstrates a smaller APD across all DI values. The slope of Tomek's APD restitution curve varies compared to TT2's, depending on the DI range (Figure 3c,d).

#### 3.1.4 Electrophysiological properties of the gray zone

**FIGURE 4:**
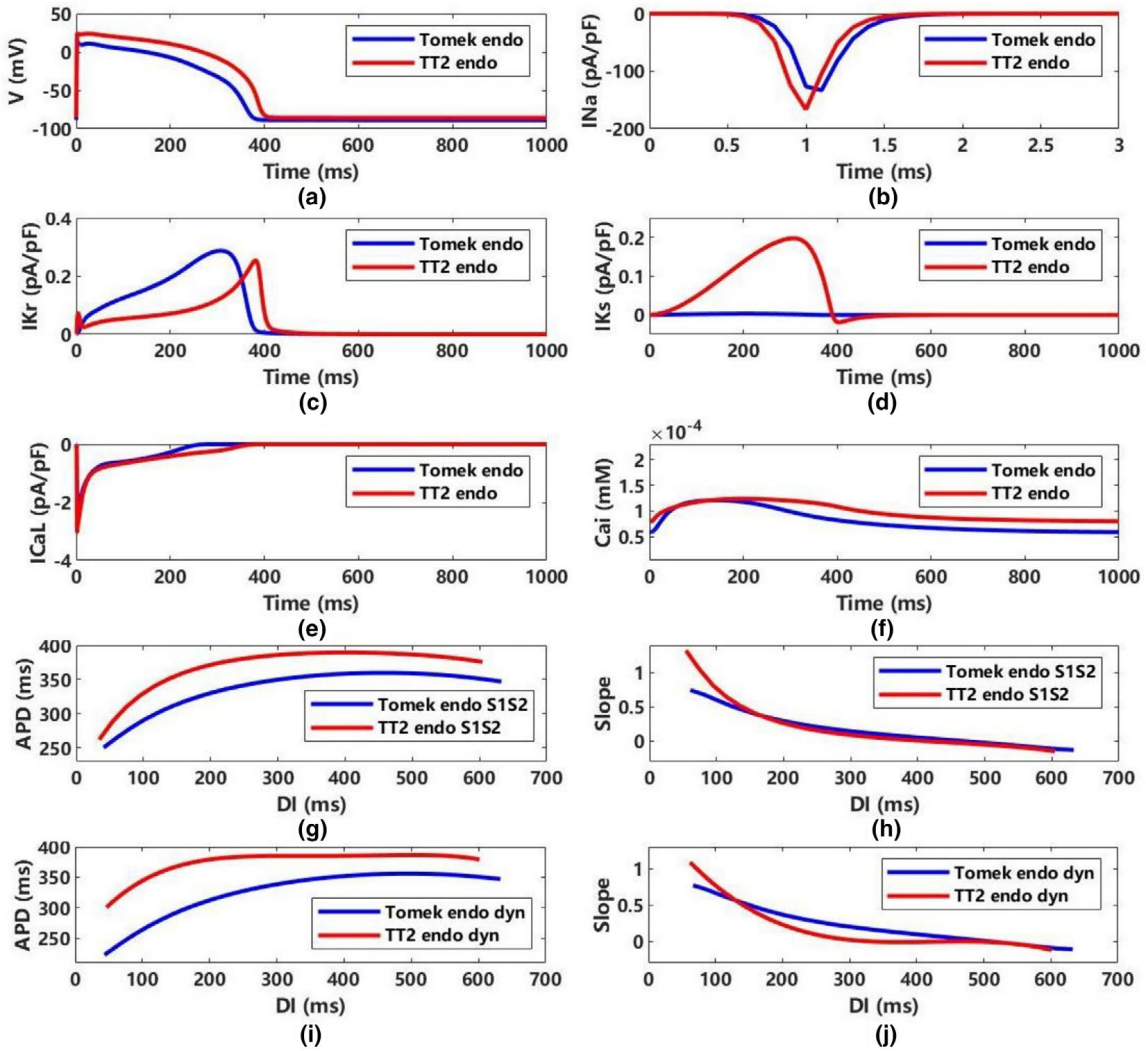
Properties of gray zone in the Tomek and the TT2 models (ENDO) with 10 μs timestep. (a) membrane potentials, (b‐f) major ion currents modified in the model of gray zone, including I_Na_, I_Kr_, I_Ks_, I_K1_, I_CaL_, and [Ca^2+^]_i_, (G) APD restitution curves in S1‐S2 protocol, (H) the slope of APD restitution curves in S1‐S2 protocol, (i) APD restitution curves in dynamic protocol, (j) the slope of APD restitution curves in dynamic protocol. APD represents APD90; DI, diastolic interval; dyn, dynamic protocol; ENDO, endocardial; S1S2, S1‐S2 protocol.

Figure 4 illustrates the action potential and key ion currents for the Tomek and TT2 models in the gray zone; additional details for endocardial and middle cells are provided in Figures S5 and S6. The TT2 model has a higher peak voltage (23.343 mV vs. 14.761 mV in Tomek), a longer APD (392.02 ms vs. 366.21 ms), and a less negative resting potential (−85.831 mV vs. −88.847 mV). Table 2 lists the values of various ion currents in the gray zone between the Tomek and TT2 models.

- INa: Smaller in Tomek (−133.028 pA/pF) compared to TT2 (−166.714 pA/pF).
- IKr: TT2 presents a narrow early spike with slightly lower magnitude (0.253 pA/pF vs. 0.287 pA/pF).
- IKs: Larger in TT2 (0.1974 pA/pF vs. 0.0031 pA/pF).
- ICaL: Larger in TT2 (−3.033 pA/pF) compared to Tomek (−2.242 pA/pF), and it inactivates more rapidly.
- [Ca^2+^]_i_: Higher in TT2 (0.1237 μM vs. 0.1209 μM), and remains elevated for a longer portion of the recovery phase.

**TABLE 2:** Peak value of different ion currents in the gray zone between TT2 and Tomek models.

Model	INa(pA/pF)	I_Kr_(pA/pF)	I_Ks_(pA/pF)	I_CaL_(pA/pF)	[Ca2+]i(μM)
TT2	−166.714	0.253	0.1974	−3.033	0.1237
Tomek	−133.028	0.287	0.0031	−2.242	0.1209

#### 3.1.5 APD restitution in the gray zone

Figure 4g,h depict APD restitution curves using S1–S2. Tomek's APD is smaller than TT2's for all DI values (Figure 4g). Their slopes are similar when DI > 140 ms (Figure 4h). Figure 4i,j show the dynamic APD restitution curves and slopes, revealing that Tomek's APD is consistently lower than TT2's (Figure 4i), and the difference increases with smaller DI. Tomek's APD slope surpasses TT2's when DI > 130 ms (Figure 4j).

#### 3.1.6 CV restitution in normal and infarct tissue

**FIGURE 5:**
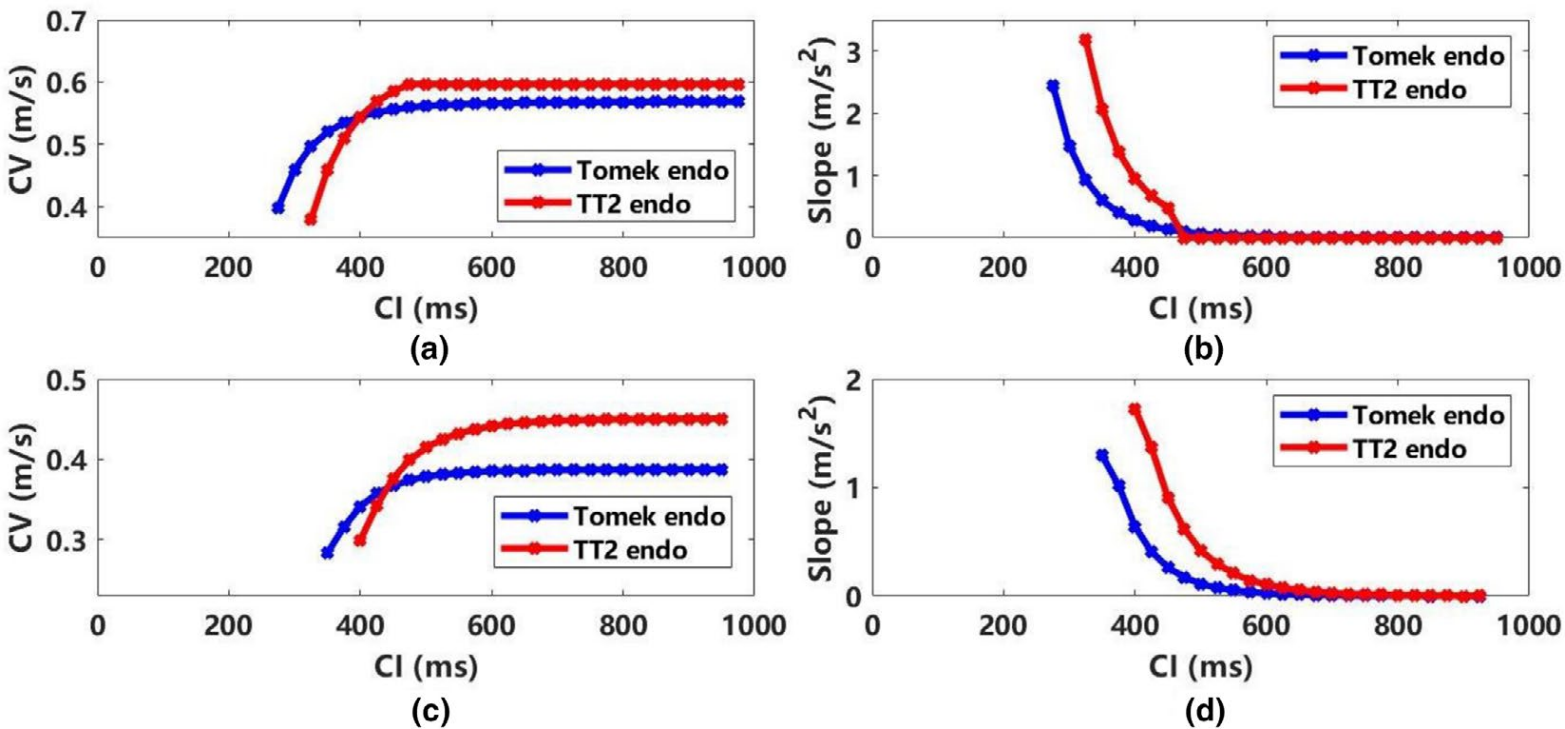
CV restitution curves and its slope of normal and infarct tissue in the Tomek and the TT2 models (ENDO) measured in a 1D cable model with 10 μs timestep. (a) CV restitution curve of normal tissue; (b) the slope of CV restitution curve in normal tissue; (c) CV restitution curve of infarct tissue; (d) the slope of CV restitution curves in infarct tissue. CI, coupling interval; CV, conduction velocity; ENDO, endocardial.

Figure 5 compares conduction velocity (CV) in normal and infarct tissue; additional details for endocardial and middle cells are provided in Figures S7 and S8. In normal tissue (Figure 5a,b), the TT2 model shows a faster CV than Tomek for CI > 400 ms, with a steeper restitution slop for CI < 475 ms. Both models stabilize when CI > 500 ms. In infarct tissue (Figure 5c,d), the TT2 maintains faster CV and steeper slopes, but both models converge when CI > 650 ms.

### 3.2 Electrophysiological characterization of the Tomek and TT2 models at the tissue level

Previous research has shown that uniform tetrahedral meshes result in larger relative differences in activation time compared to adaptive meshes (Cao et al., 2023). Due to their superior stability, adaptive tetrahedral meshes were used for all 2D simulations in this study.

#### 3.2.1 Reentry dynamics in the Tomek model

**FIGURE 6:**
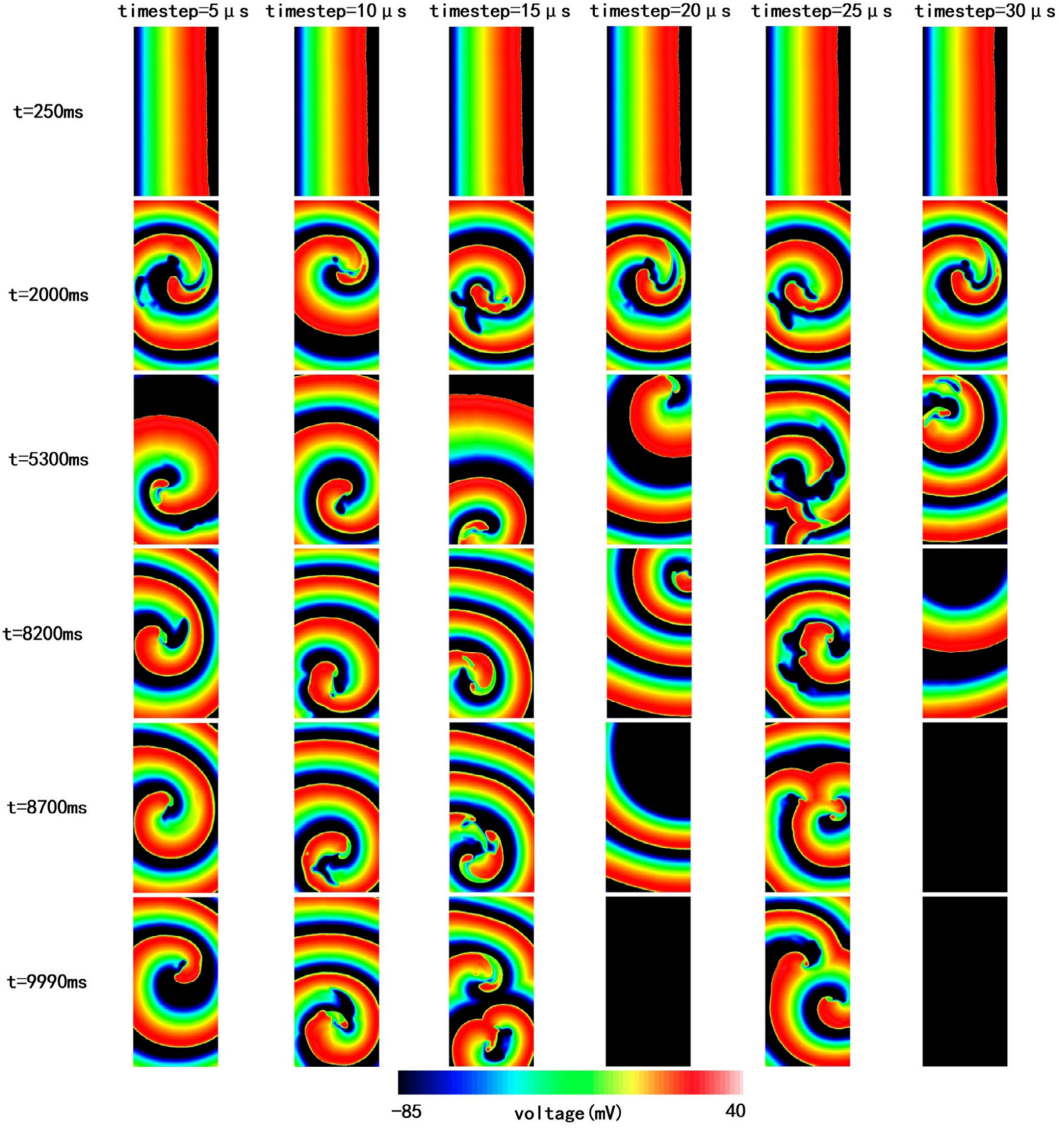
The electrical propagation at simulation time of 250, 2000, 5300, 8200, 8700, and 9990 ms with 5, 10, 15, 20, 25, and 30 μs timesteps, respectively, with the Tomek model (endocardium).

In the Tomek model, reentry behavior varied across time steps (5–30 μs, Video S1). Figure 6 illustrates the electrical propagation at six different moments during simulation. At a 20 μs time step, reentry disappeared in the top‐right corner of the grid at approximately 8600 ms. At 30 μs, propagation vanished in the top‐left corner at around 8200 ms. In contrast, reentries persisted for the entire 10‐s simulation at 5, 10, 15, and 25 μs.

Reentry morphology and location showed no consistent pattern across time steps and wave fragmentation occurred in some cases (e.g., 15 μs and 25 μs), resulting in multiple concurrent reentries. The premature terminations and wave breaks may be influenced by boundary effects, which could potentially be mitigated by increasing the mesh size.

#### 3.2.2 Reentry dynamics in the TT2 model

**FIGURE 7:**
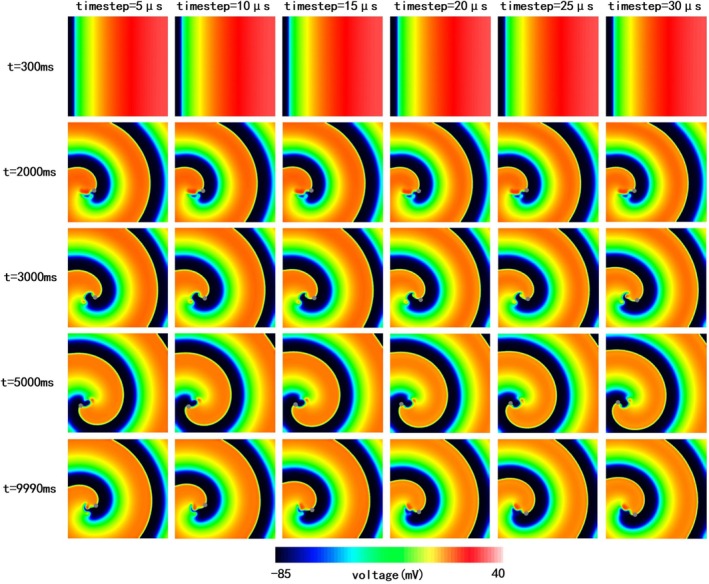
The electrical propagation at simulation times of 300, 2000, 3000, 5000, and 9990 ms with 5, 10, 15, 20, 25, and 30 μs timesteps respectively with the TT2 model (endocardium).

In contrast to the Tomek model, the TT2 model showed no significant differences in reentry morphology or location for time steps between 5 μs and 30 μs (Figure 7 and Video S2). No wave breaks were observed, and reentry morphologies and phase singularity locations remained stable throughout the simulation. Only minor variations in voltage distribution near the reentry tip were noted toward the end of the simulation (*t* = 9990 ms). These findings suggest that the TT2 model is less sensitive to changes in time step in 2D tissue simulations.

#### 3.2.3 Comparison of reentry dynamics (Tomek vs. TT2)

**FIGURE 8:**
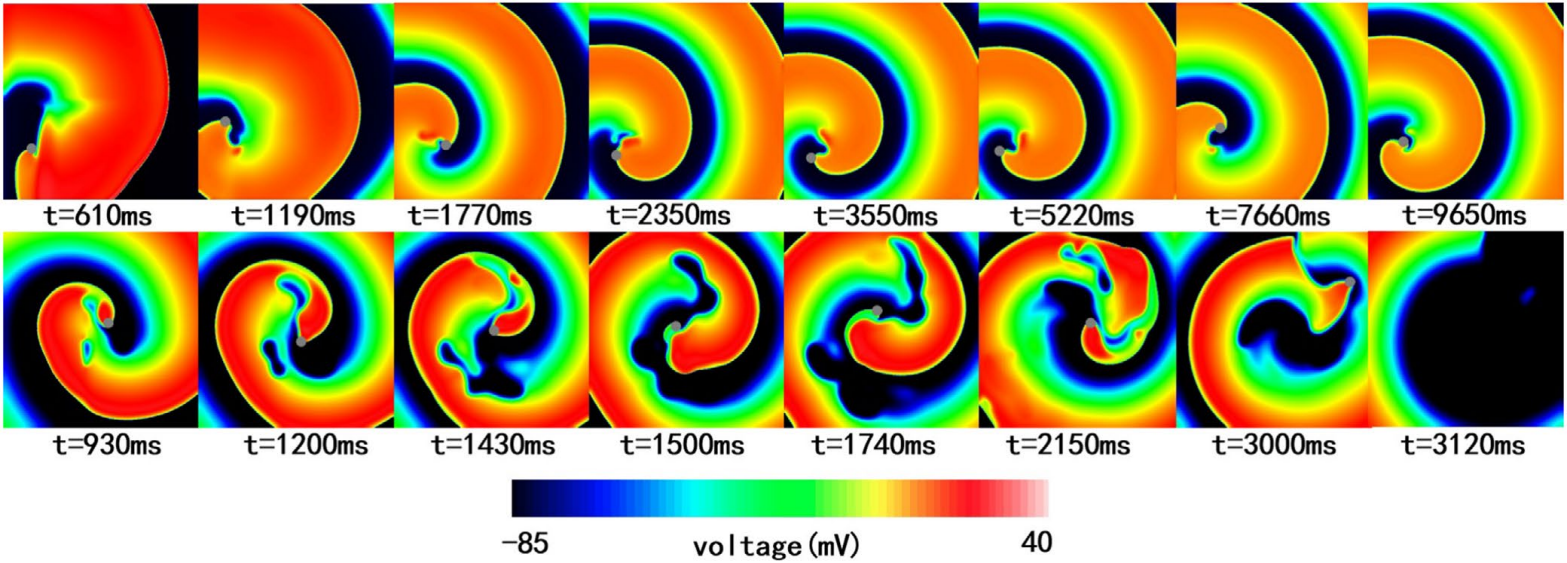
Reentry simulation in 2D tissue with the TT2 and the Tomek model with 10 μs timestep. The first row showed the reentry induced with the TT2 model at different simulation times. The second row was the reentry induced with the Tomek model at different simulation times.

Figure 8 compares the reentry dynamics of the two models. The TT2 model demonstrated stable reentry forms and reentry center positions (see Figure 8, first row; Video S2), while the Tomek model showed multiple wave breaks and more chaotic reentries, with the center shifting from the tissue core to the right edge (Figure 8, second row; Video S2).

#### 3.2.4 [Ca^2+^]_i_ analysis

**FIGURE 9:**
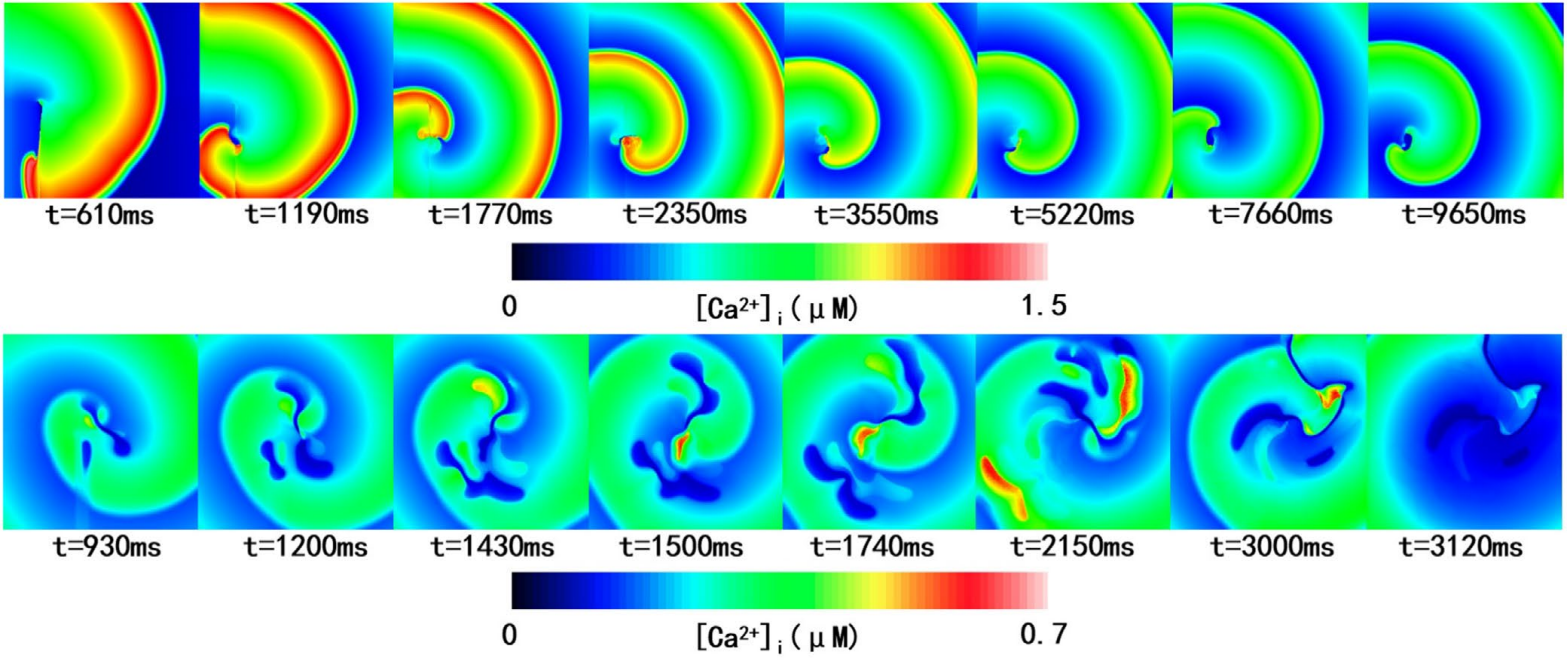
[Ca^2+^]_i_ in 2D tissue with the TT2 and the Tomek model with a 10 μs timestep. The first row shows [Ca^2+^]_i_ with the TT2 model at different simulation times. The second row was [Ca^2+^]_i_ with the Tomek model at different simulation times.

Differences in [Ca^2+^]_i_ dynamics were analyzed to explore the instability of reentries in the Tomek model (Figure 9; Videos S3 and S4). In the TT2 model (10 μs time step; Figure 9, first row; Video S3), [Ca^2+^]_i_ stabilized after initial cycles, correlating with the consistent reentry patterns observed in the model.

Conversely, in the Tomek model (10 μs time step; Figure 9, second row; Video S4), pronounced spatial heterogeneity in [Ca^2+^]_i_ was observed, with wave breaks and shifting reentry centers prevalent. The heterogeneity likely contributes to the chaotic reentry behavior seen in the Tomek model.

#### 3.2.5 Gray zone tissue simulations

**FIGURE 10:**
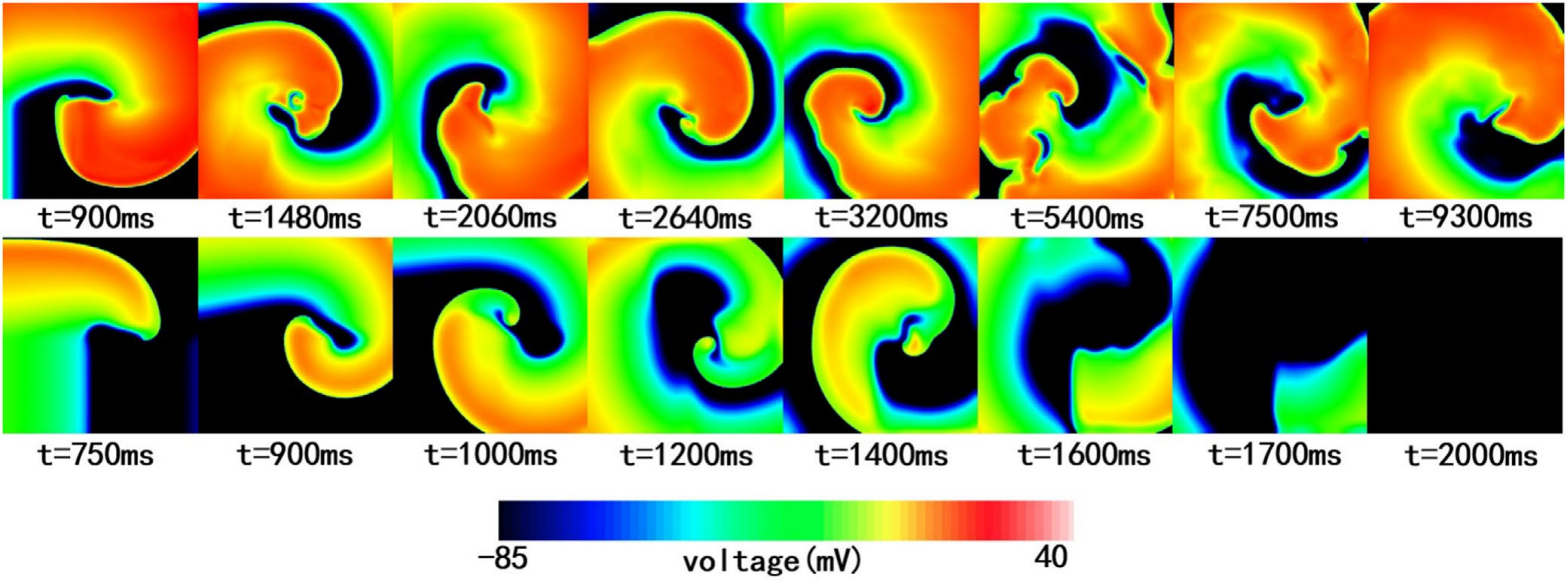
Reentry simulation in 2D tissue using the TT2 and Tomek gray zone models with a 10 μs time step. The first row shows reentry patterns induced by the TT2 gray zone model at various simulation times. The second row displays reentry patterns induced by the Tomek gray zone model at corresponding simulation times.

Figure 10 shows reentry morphologies in gray zone tissue at different time steps. In the TT2 model (Figure 10, first row; Video S5), reentry centers remained relatively stable throughout the simulation. In contrast, the Tomek gray zone tissue model (Figure 10, second row; Video S6) experienced premature reentry termination at approximately 1700 ms, accompanied by a lower membrane potential compared to TT2.

### 3.3 Electrophysiological properties of the Tomek and TT2 models at the organ level

#### 3.3.1 Computation time

On a system with an Intel® Core™ i7‐8700K CPU @ 3.70 GHz, patient‐specific ventricular simulations using the Tomek model required approximately 1.5 times more computation time than the TT2 model (time step = 25 μs). Similarly, in single‐cell simulations at 10 μs, the Tomek model took ~1.7 times longer than TT2.

#### 3.3.2 Induced reentries in the patient‐specific ventricular model

Three reentry morphologies were induced at s4 in simulations with the TT2 model

**FIGURE 11:**
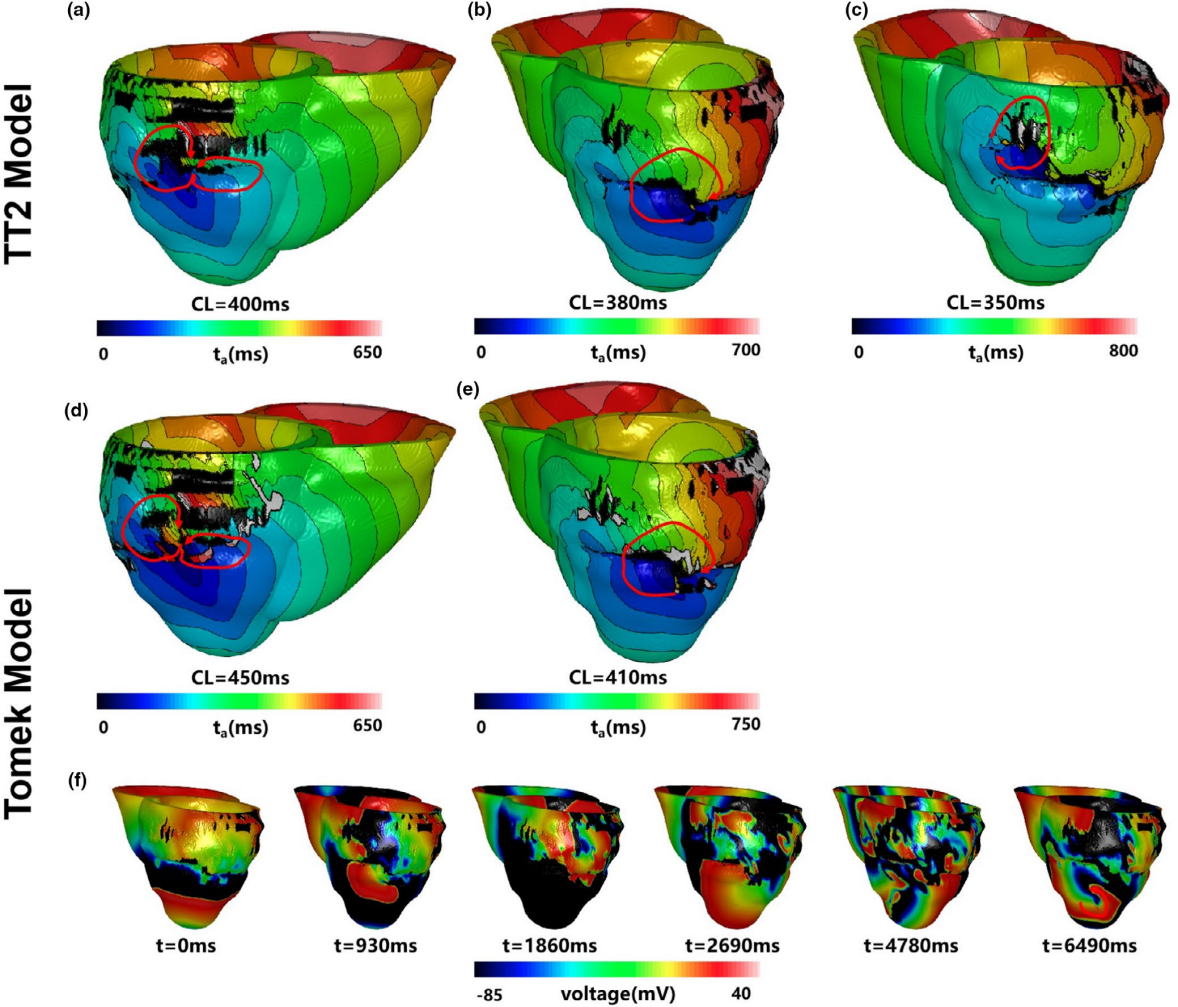
The three types of reentries induced in patient‐specific simulation with the TT2 and the Tomek models. (a–c) showed the activation maps of the TT2 model, and (d) and (e) showed the activation maps of the Tomek model. (f) showed an image of the membrane potential change of the Tomek model, whose reentry pattern began in a chaotic state but finally stabilized. (a) The reentry induced at the posterior wall with the TT2 model; (b) the reentry induced at the anterior lateral wall with the TT2 model; (c) the reentry only induced in the TT2 model, which was not found in the Tomek, was in the upper front wall; (d) the reentry induced at the posterior wall with the Tomek model; (e) the reentry induced at the anterior lateral wall with the Tomek model; (f) the unique reentry only induced in the Tomek model, which was not found in TT2, was in the lower part of the anterior wall.

1. One at the posterior wall (Figure 11a; Video S7)
2. One at the anterior lateral wall (Figure 11b; Video S8)
3. A unique reentry in the upper front wall (Figure 11c; Video S9) that did not appear in the Tomek model

Similarly, three reentries were induced at s2 in the Tomek model

1. One at the posterior wall (Figure 11d; Video S10)
2. One at the anterior lateral wall (Figure 11e; Video S11)
3. A unique reentry in the lower anterior lateral wall (Figure 11f; Video S12), which began chaotically before stabilizing in the anterior lateral region

**FIGURE 12:**
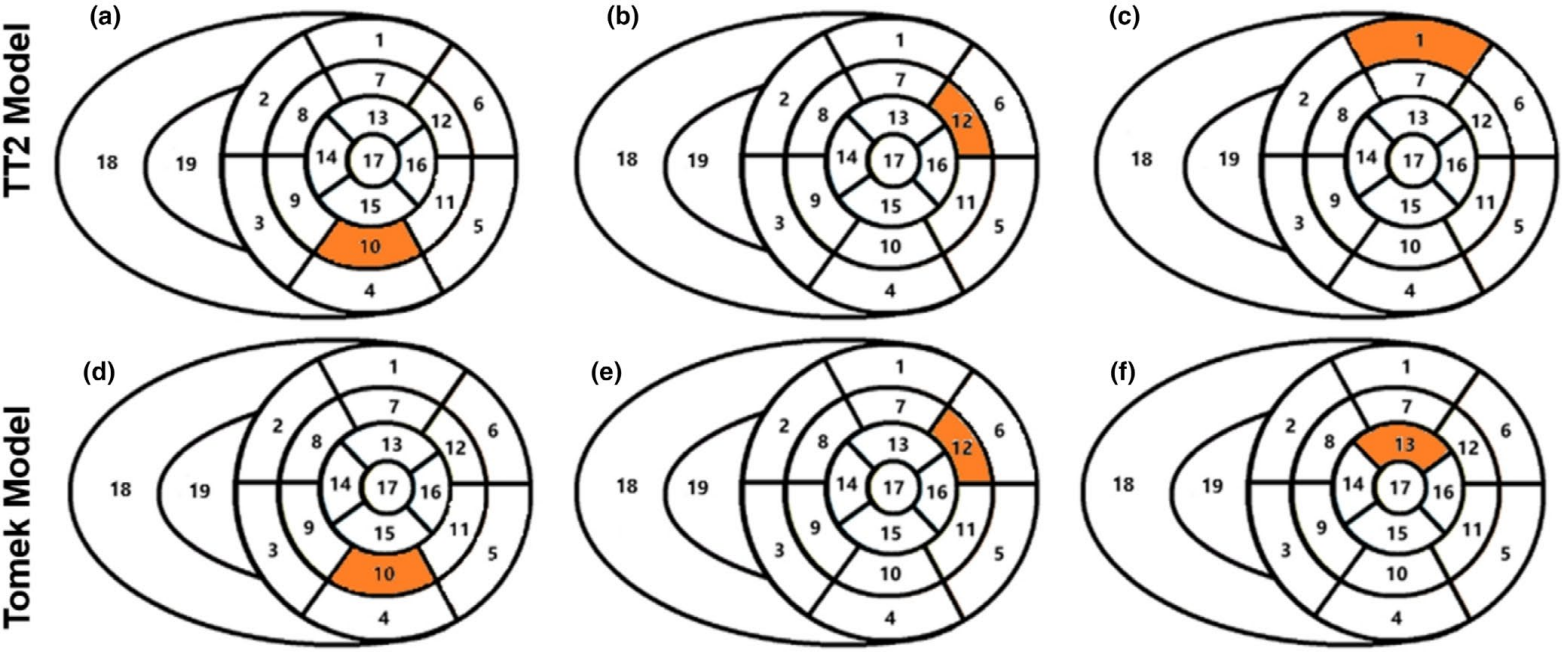
The reentries' location induced in patient‐specific simulation with the TT2 and the Tomek models. (a) The reentry induced at the posterior wall with the TT2 model; (b) the reentry induced at the anterior lateral wall with the TT2 model; (c) the reentry only induced in the TT2 model, which was not found in the Tomek, was in the upper front wall; (d) the reentry induced at the posterior wall with the Tomek model; (e) the reentry induced at the anterior lateral wall with the Tomek model; (f) the unique reentry only induced in the Tomek model, which was not found in TT2, was in the lower part of the anterior wall.

Table 3 shows the Reentry types induced by the TT2 and the Tomek individualized 3D models with the time steps ranged from 5 to 30 μs. Figure 12 highlighted differences in reentry locations between the TT2 and Tomek models. The reentry cycle lengths were slightly longer in the Tomek model compared to the TT2 model (e.g., 450 ms vs. 400 ms, 410 ms vs. 380 m).

**TABLE 3:** Reentry types induced by the TT2 and the Tomek individualized 3D models with the time steps ranged from 5 to 30 μs. Segment XX represented the reentry location induced in the corresponding simulation models according to the AHA 17 segments.

Time steps (μs)	Segment 1		Segment 10		Segment 12		Segment 13	
	TT2	Tomek	TT2	Tomek	TT2	Tomek	TT2	Tomek
5	**√**	**×**	**√**	**√**	**√**	**√**	**×**	**√**
10	**√**	**×**	**√**	**√**	**√**	**√**	**×**	**×**
15	**√**	**×**	**√**	**√**	**√**	**√**	**×**	**×**
20	**√**	**×**	**√**	**√**	**√**	**√**	**×**	**×**
25	**√**	**×**	**√**	**√**	**√**	**√**	**×**	**×**
30	**√**	**×**	**√**	**√**	**√**	**√**	**×**	**×**

#### 3.3.3 Effect of time step

**FIGURE 13:**
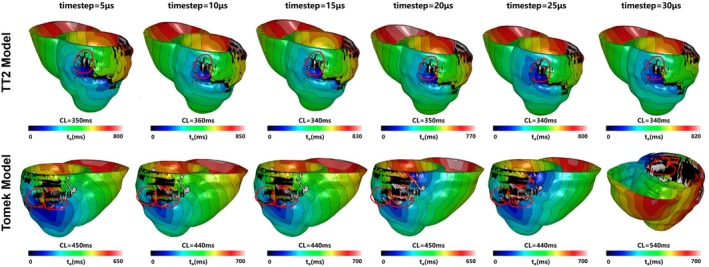
The two reentries induced in patient‐specific simulation with the TT2 and the Tomek models with the time steps ranged from 5 to 30 μs. The first row showed the reentry induced at the anterior lateral wall with the TT2 model. The second row showed the reentry induced at posterior wall with the Tomek model. CL, cycle length.

Reentry behaviors in the TT2 model were consistent across time steps from 5 to 30 μs (Figure 13, first row). Morphologies, locations, and reentry types (segments 1, 10, and 12 based on the American Heart Association Classification (Cerqueira et al., 2002)) showed minor variations, with only minor differences in cycle lengths. This demonstrates the TT2 model's robustness to changes in time step at the organ level.

In the Tomek model (Figure 13, second row), reentry types (segments 10, 12, and 13) remained stable from 5 to 25 μs, with minor cycle length variations. However, at 30 μs, the reentry center shifted closer to the boundary, likely due to artifacts introduced by the larger time step. This sensitivity aligns with the 2D results, indicating that the Tomek model is more sensitive to time‐step variations than TT2.

## 4 DISCUSSION

This study compared the electrophysiological properties of two human ventricular cell models—Tomek and TT2—across single cell (0D), linear cable model (1D), tissue (2D), and organ (3D) scales. Both models capture key features of human ventricular action potentials and ion currents, but notable differences emerged in action potential morphology, APD restitution, conduction velocity (CV), and reentrant activity. These discrepancies reflect each model's formulation assumptions and the extent of its updated experimental data and detailed calcium‐handling mechanisms.

At the single‐cell level, the TT2 model demonstrated stability over time steps ranging from 5 to 30 μs, consistent with a previous finding that the TT2 model performs reliably under relatively large time steps (Cooper et al., 2015). The Tomek model showed comparable stability, despite incorporating more detailed formulations of ion channel and calcium dynamics.

At the tissue level, the TT2 model, with its distinct ionic current profile and calcium dynamics, produced more stable reentry patterns in 2D. Conversely, the Tomek model exhibited more chaotic reentries and wave breaks, particularly under the same stimulation protocol. This difference is likely attributed to the stronger spatial heterogeneity in intracellular calcium ([Ca2+]i) modeled in the Tomek model. While this detailed calcium handling enhances physiological realism at the cellular scale, it may introduce additional instability in tissue‐level simulations unless mesh resolution and parameter tuning are carefully managed.

In patient‐specific 3D simulations, both models generated qualitatively similar reentrant morphologies, particularly in the posterior and anterior lateral walls. Despite differences in reentry cycle lengths, both models reproduced the post‐infarct VT mechanism—unidirectional block and reentry within the gray zone (Amoni et al., 2021; Emami et al., 2019; Segal et al., 2010). Each model generated one unique reentrant circuit, reflecting slight differences in conduction velocity and ion current formulations. However, these variations did not impact the identification of critical conduction channels relevant for ablation in scar‐related VT (Martin et al., 2018; Nishimura et al., 2021).

A further distinction lies in the number of extra‐stimuli required to induce reentry. The TT2 model often required only one premature beat, whereas the Tomek model frequently required up to three. Clinically, up to three extra‐stimuli are used to reach the effective refractory period and induce VT (Fernández‐Armenta et al., 2016), suggesting that both models align with realistic protocols. Nonetheless, the Tomek model's time‐step sensitivity was more obvious, especially in 2D simulations where a 30 μs time step occasionally generated nonclinical “pseudo‐reentries.” Smaller time steps or additional calibration may thus be necessary for large‐scale simulations using the Tomek model.

In terms of computational efficiency, the Tomek model required approximately 1.5–1.7 times longer compute time than TT2, likely due to its more detailed ionic current formulations. Though the TT2 model relies on older experimental data (ten Tusscher et al., 2004), is remains well‐validated for reentrant phenomena in clinical studies (O'Hara et al., 2021; Prakosa et al., 2018; Shade et al., 2021; Tong et al., 2021). By contrast, the Tomek model includes further refinements to ion currents and is more easily to get induced functional reentries (Ikeda et al., 1996; Iravanian et al., 2003), which may make it better suited for exploring complex arrhythmogenic mechanisms or drug interactions. The TT2 model's advantage lies in its quicker run times and straightforward Hodgkin–Huxley‐type structure, making it preferable for large‐scale studies requiring computational efficiency.

Overall, our findings indicate that both the Tomek and TT2 models are valuable tools for simulating post‐infarct VT, each offering distinct advantages depending on the simulation goals. The TT2 model, characterized by its Hodgkin‐Huxley‐type formulation and demonstrated numerical stability across a wide range of time steps, is well‐suited for large‐scale 3D simulations and investigations prioritizing computational efficiency, particularly in studies of canonical reentrant arrhythmias. In contrast, the Tomek model incorporates more detailed experimental data on human ion channel kinetics and calcium dynamics, resulting in enhanced physiological fidelity. This makes it a strong choice for mechanistic investigations into calcium‐driven arrhythmogenesis, drug testing, and studies focused on subtle ionic or molecular alterations. However, its added complexity leads to greater computational cost—approximately 1.5× longer simulation times—and increased sensitivity to time step and mesh resolution. This can introduce numerical instability in 2D/3D tissue simulations if not careful managed.

In summary, while TT2 provides a robust and efficient framework for large‐scale functional simulations, the Tomek model is better suited for detailed mechanistic studies. These complementary strengths underscore the importance of selecting a model aligned with research focus, balancing computational feasibility with physiological accuracy.

Future efforts might explore hybrid approaches, such as integrating the updated ionic formulations from the Tomek model into the more computationally efficient Hodgkin–Huxley structure of TT2. Additionally, simplifying selectively removing redundant Ca2 + −signaling components—as proposed by O'Hara et al. (2011)—could further reduce computational burden without compromising essential electrophysiological fidelity.

There are, however, limitations to this study. The 2D mesh size used for the Tomek model was sometimes insufficient to prevent boundary‐related reentry termination, indicating that larger meshes may be required. Although only one patient‐specific heart model was studied (which demonstrated certain differences) and we assessed only five sites known to induce reentry in the TT2 model for the Tomek model organ‐level simulation, a clinical case‐series retrospective study involving both models could be undertaken. Despite these constraints, this comparative analysis emphasizes the importance of model selection in influencing VT stability, cycle length, and computational requirements, providing important insights for both mechanistic studies and clinical translation.

## 5 CONCLUSIONS

In this study, we compared the electrophysiological properties of the TT2 model with the Tomek model at the cellular, tissue, and organ levels. We found that the TT2 model showed distinct action potential and main ion current morphologies compared to the Tomek model, leading to significant differences in the APD and CV restitution curves, as well as in the reentrant behavior observed in 2D tissue simulations. Our preliminary 3D findings indicate that both models are capable of reproducing key features of post‐infarct ventricular tachycardia (VT). However, given that these organ‐level simulations were based on a single patient‐specific model and limited stimulation sites for one of the models, further validation with a larger cohort of patient‐specific models and broader clinical data is necessary to definitively establish their suitability and comparative efficacy for predicting clinical VT characteristics. Nevertheless, this study highlights that the choice of cellular model influences simulation outcomes across spatial scales. Recognizing the respective strengths and limitations of each model will be critical for guiding future computational studies in cardiac arrhythmia research.

## AUTHOR CONTRIBUTIONS

R.D, C.Z, Z.F, S.J, and D.D were responsible for the conception or design of the work. C.Z, Z.F, Y.L, Y.W, N.Z, Z.W, J.H, L.X, and Y.W were responsible for the acquisition, analysis, or interpretation of data for the work. R.D, C.Z, Z.F, S.J, and D.D were responsible for drafting the work or revising it critically for important intellectual content. R.D, C.Z, Z.F, Y.L, Y.W, N.Z, Z.W, J.H, L.X, Y.W, S.J, and D.D were responsible for final approval of the version to be published.

## ETHICS STATEMENT

This research does not involve sensitive data or materials that require specific ethical approval. All procedures were conducted in line with general ethical guidelines for research, ensuring integrity and responsibility in data collection, analysis, and reporting.

Data used in this study were either publicly available, anonymized, or obtained through non‐invasive means with appropriate permissions. No personal or confidential information was involved, and all data handling practices adhered to standard principles of data protection and privacy.

The research team maintained a commitment to ethical conduct throughout, with a focus on accuracy, transparency, and avoiding any potential harm or misrepresentation.

## Supporting information


Appendix S1.

## Data Availability

All relevant data are included within the paper and Appendix S1. The mesh and simulation files that support the findings of this study are available from the corresponding author upon reasonable request.
