# Supplementary material for: Comparative analysis of the ten Tusscher and Tomek human ventricular cell models at cellular, tissue, and organ levels: Implications for post‐infarct ventricular tachycardia simulation
Source: Physiol Rep. 2025 Jul 10;13(13):e70435. doi: 10.14814/phy2.70435 (PMC12246384; doi:10.14814/phy2.70435)
Supplement: Supplementary file 1 — Appendix S1. [file PHY2-13-e70435-s001.zip › Appendix.docx]

**Appendix**

**Comparative Analysis of the ten Tusscher and Tomek Human Ventricular Cell Models at Cellular, Tissue, and Organ Levels: Implications for Post-Infarct Ventricular Tachycardia Simulation**

Ruiqing Dong1#, Zhenyin Fu2#, Chuxin Zhang3#, Yumeng Liu4, Yiming Wang3, Nan Zhang5, Zefeng Wang6, Jun Hou1, Ling Xia2, Yongquan Wu6*, Shijie Zhou7*, Dongdong Deng3*

**Ion Currents and Action Potential Morphology of epicardium cell**

FigureA1 compares action potentials and major ion currents between the Tomek and TT2 models of epicardium cell. The TT2 model has a higher peak action potential (~40.68 mV) and a longer APD (306.74 ms) than the Tomek model (~33.18 mV, 244.89 ms). The Tomek model shows a more negative resting potential (-88.88 mV vs. -85.41 mV), attributed to differences in IK1 currents.

Table S1 illustrates quantitative differences in major ion currents between the two models.

- INa (Figure S1B): The Tomek model activates INa later and with a smaller peak magnitude than the TT2 model (-295.2 pA/pF vs. -325.7 pA/pF).
- Ito (Figure S1C): The transient outward current is larger in the Tomek model (11.12 pA/pF vs. 3.73 pA/pF).
- IKr (Figure S1D): The TT2 model shows a narrow early spike of IKr with a smaller amplitude than in the Tomek model (0.8154 pA/pF vs. 1.264 pA/pF).
- IKs (Figure S1E): The TT2 model exhibits a much larger IKs (0.9176 pA/pF vs. 0.02299 pA/pF in Tomek).
- IK1 (Figure S1F): Both models have two peaks in IK1, but the TT2 model’s magnitude is larger (1.835 pA/pF vs. 1.322 pA/pF).
- INaK (Figure S1G): The TT2 model has a smaller INaK peak than Tomek (0.363 pA/pF vs. 0.4861 pA/pF).
- ICaL (Figure S1H): TT2 exhibits a larger peak ICaL than Tomek (-5.185 pA/pF vs. -7.7 pA/pF) and decreases faster, followed by a plateau phase.
- INaCa,i (Figure S1I): The TT2 model includes only the bulk myoplasm component, whereas Tomek divides INaCa into junctional subspace and bulk myoplasm, leading to distinct waveforms.
- [Ca2+]i (Figure S1J): The TT2 model has higher intracellular calcium levels (0.7612 μM vs. 0.6791 μM in Tomek).


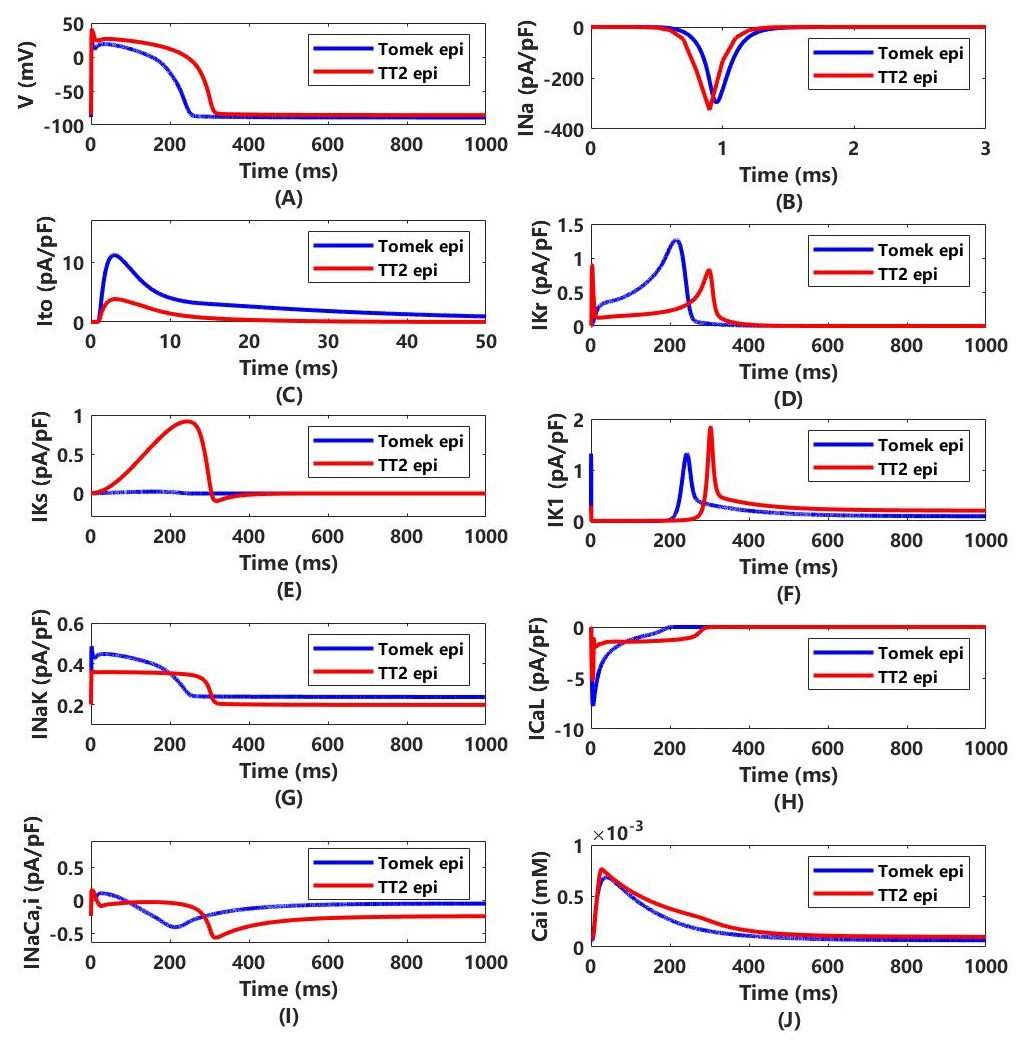


**Figure S1.** The morphologies of the action potential and major ion currents in the Tomek and the TT2 models (EPI) with 10 µs timestep. (A) The membrane potentials, (B-J) major ion currents, including I_Na_, I_to_, I_Kr_, I_Ks_, I_K1_, I_NaK_, I_CaL_, I_NaCa,i_ and [Ca^2+^]_i_. EPI: epicardial.

**Table S1.** Peak Value of Different Ion Currents between TT2 and Tomek models of epi cell

| **Model** | **INa(pA/pF)** | **I_to_(pA/pF)** | **I_Kr_(pA/pF)** | **I_Ks_(pA/pF)** | **I_k1_(pA/pF)** | **I_NaK_(pA/pF)** | **I_CaL_(pA/pF)** | **[Ca2+]i(μM)** | **INaCa,i** |
| --- | --- | --- | --- | --- | --- | --- | --- | --- | --- |
| TT2 | -325.7 | 3.73 | 0.8154 | 0.9176 | 1.835 | 0.363 | -5.185 | 0.7612 | Bulk myoplasm component |
| Tomek | -295.2 | 11.12 | 1.264 | 0.02299 | 1.322 | 0.4861 | -7.7 | 0.6791 | Junctional subspace + bulk myoplasm |

**Ion Currents and Action Potential Morphology of middle cell**

FigureA2 compares action potentials and major ion currents between the Tomek and TT2 models of middle cell. The TT2 model has a higher peak action potential (~40.68 mV) and a longer APD (410.38 ms) than the Tomek model (~31.67 mV, 347.83 ms). The Tomek model shows a more negative resting potential (-88.5 mV vs. -85.41 mV), attributed to differences in IK1 currents.

Table S2 illustrates quantitative differences in major ion currents between the two models.

- INa (Figure S2B): The Tomek model activates INa later and with a smaller peak magnitude than the TT2 model (-281 pA/pF vs. -325.7 pA/pF).
- Ito (Figure S2C): The transient outward current is larger in the Tomek model (10.16 pA/pF vs. 3.822 pA/pF).
- IKr (Figure S2D): The TT2 model shows a narrow early spike of IKr, but the magnitude was similar with the Tomek model (0.8179 pA/pF vs. 0.8049 pA/pF).
- IKs (Figure S2E): The TT2 model exhibits a much larger IKs (0.9149 pA/pF vs. 0.02133 pA/pF in Tomek).
- IK1 (Figure S2F): Both models have two peaks in IK1, but the TT2 model’s magnitude is larger (1.835 pA/pF vs. 1.432 pA/pF).
- INaK (Figure S2G): The TT2 model has a smaller INaK peak than Tomek (0.363 pA/pF vs. 0.5226 pA/pF).
- ICaL (Figure S2H): TT2 exhibits a larger peak ICaL than Tomek (-5.185 pA/pF vs. -10.28 pA/pF) and decreases faster, followed by a plateau phase.
- INaCa,i (Figure S2I): The TT2 model includes only the bulk myoplasm component, whereas Tomek divides INaCa into junctional subspace and bulk myoplasm, leading to distinct waveforms.
- [Ca2+]i (Figure S2J): The TT2 model has smaller intracellular calcium levels (0.7609 μM vs. 1.076 μM in Tomek).


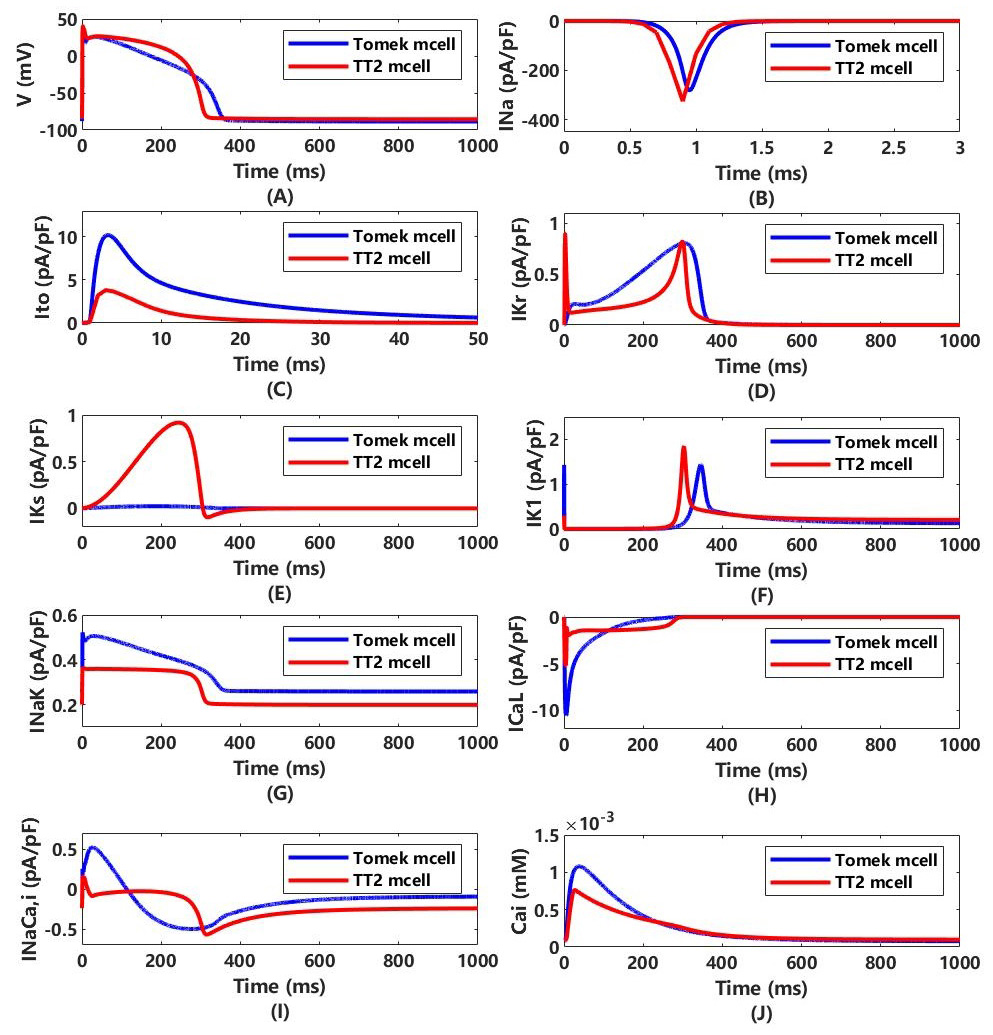


**Figure S2.** The morphologies of the action potential and major ion currents in the Tomek and the TT2 models (MCELL) with 10 µs timestep. (A) The membrane potentials, (B-J) major ion currents, including I_Na_, I_to_, I_Kr_, I_Ks_, I_K1_, I_NaK_, I_CaL_, I_NaCa,i_ and [Ca^2+^]_i_. MCELL: middle cell.

**Table S2.** Peak Value of Different Ion Currents between TT2 and Tomek models of middle cell

| **Model** | **INa(pA/pF)** | **I_to_(pA/pF)** | **I_Kr_(pA/pF)** | **I_Ks_(pA/pF)** | **I_k1_(pA/pF)** | **I_NaK_(pA/pF)** | **I_CaL_(pA/pF)** | **[Ca2+]i(μM)** | **INaCa,i** |
| --- | --- | --- | --- | --- | --- | --- | --- | --- | --- |
| TT2 | -325.7 | 3.822 | 0.8179 | 0.9149 | 1.835 | 0.363 | -5.185 | 0.7609 | Bulk myoplasm component |
| Tomek | -281 | 10.16 | 0.8049 | 0.02133 | 1.432 | 0.5226 | -10.28 | 1.076 | Junctional subspace + bulk myoplasm |

**APD Restitution of epicardium cell**

Figure S3 illustrates APD restitution curves under S1–S2 and dynamic protocols in the Tomek and TT2 models of epicardium cell. For the S1–S2 protocol (Figure S3A), the Tomek model’s APD is shorter than TT2’s when the diastolic interval (DI) is > 60 ms. Under the dynamic protocol (Figure S3B), the Tomek model consistently demonstrates a smaller APD across all DI values. The slope of Tomek’s APD restitution curve varies compared to TT2’s, depending on the DI range (Figure S3C, D).


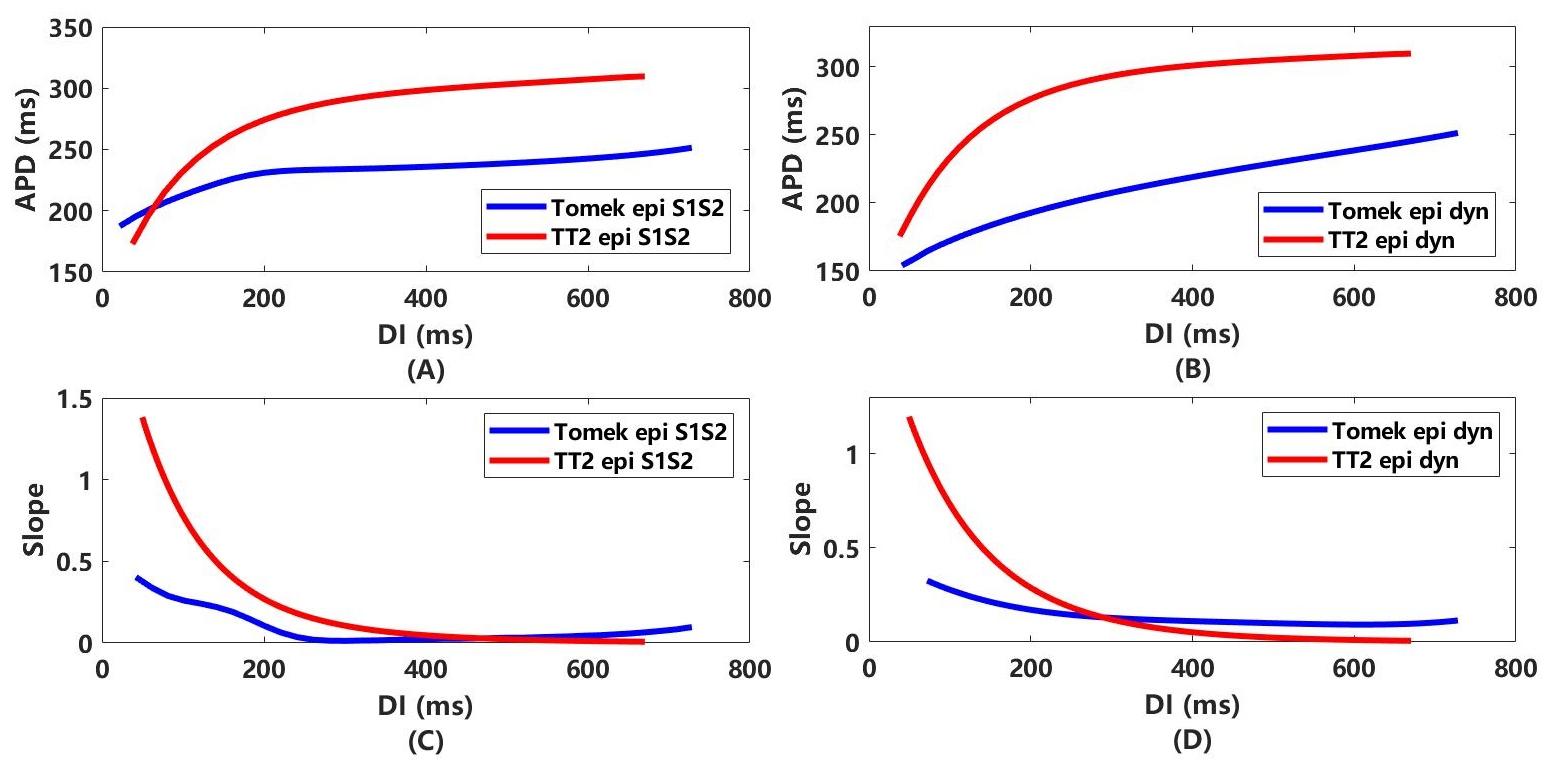


**Figure S3.** The APD restitution curves of S1-S2 and dynamic protocol in the Tomek and the TT2 models (EPI) with 10 µs timestep. (A) APD restitution curves with S1-S2 protocol in the TT2 and the Tomek models, (B) APD restitution curves with dynamic protocol in the TT2 and the Tomek models, (C) the slope of APD restitution curves with S1-S2 protocol in the TT2 and the Tomek models, (D) the slope of APD restitution curves with dynamic protocol in the TT2 and the Tomek models. APD represents APD_90_; DI: diastolic interval; S1S2: S1-S2 protocol; dyn: dynamic protocol; EPI: epicardial.

**APD Restitution of middle cell**

Figure S4 illustrates APD restitution curves under S1–S2 and dynamic protocols in the Tomek and TT2 models of middle cell. For the S1–S2 protocol (Figure S4A), the Tomek model’s APD is shorter than TT2’s when the diastolic interval (DI) is > 50 ms. Under the dynamic protocol (Figure S4B), the Tomek model consistently demonstrates a smaller APD across all DI values. The slope of Tomek’s APD restitution curve varies compared to TT2’s, depending on the DI range (Figure S4C, D).


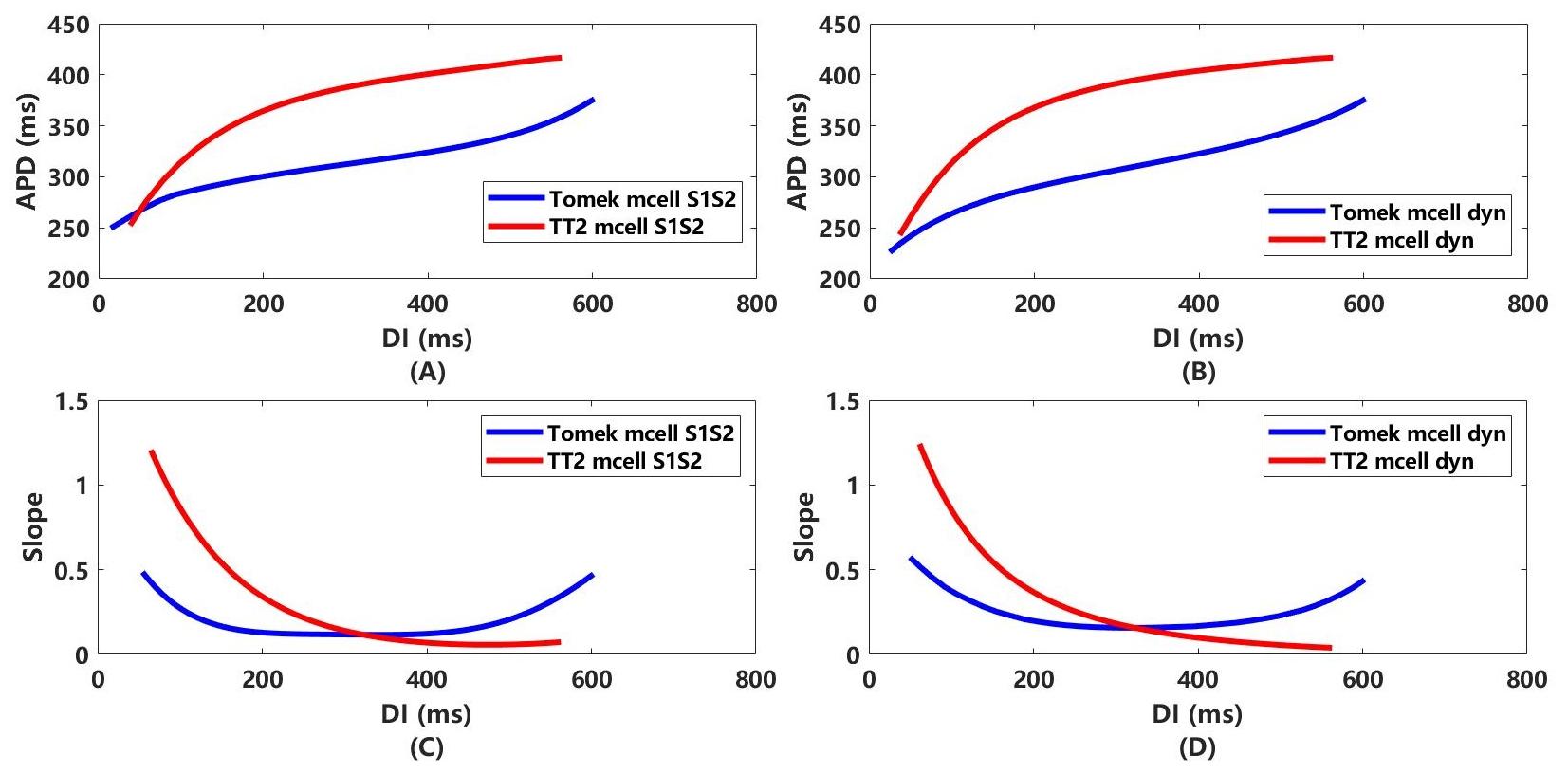


**Figure S4.** The APD restitution curves of S1-S2 and dynamic protocol in the Tomek and the TT2 models (MCELL) with 10 µs timestep. (A) APD restitution curves with S1-S2 protocol in the TT2 and the Tomek models, (B) APD restitution curves with dynamic protocol in the TT2 and the Tomek models, (C) the slope of APD restitution curves with S1-S2 protocol in the TT2 and the Tomek models, (D) the slope of APD restitution curves with dynamic protocol in the TT2 and the Tomek models. APD represents APD_90_; DI: diastolic interval; S1S2: S1-S2 protocol; dyn: dynamic protocol; MCELL: middle cell.

**Electrophysiological Properties of the Gray Zone in epicardial cell**

Figure S5 illustrates the action potential and key ion currents for the Tomek and TT2 models in the gray zone of epicardial cell. The TT2 model has a higher peak voltage (21.70 mV vs. 13.94 mV in Tomek), a longer APD (384.86 ms vs. 346.32 ms), and a less negative resting potential (-85.85 mV vs. -88.94 mV). Table S3 lists the values of various ion currents in the gray zone between the Tomek and TT2 models.

- INa: Smaller in Tomek (-135.257 pA/pF) compared to TT2 (-166.892 pA/pF).
- IKr: TT2 presents a narrow early spike with slightly lower magnitude (0.252 pA/pF vs. 0.354 pA/pF).
- IKs: Significantly higher in TT2 (0.1818 pA/pF vs. 0.0043 pA/pF).
- ICaL: Larger in TT2 (-3.608 pA/pF) compared to Tomek (-2.985 pA/pF), and it inactivates more rapidly.
- [Ca^2+^]_i_: Higher in TT2 (0.1219 μM vs. 0.1138 μM), and remains elevated for a longer portion of the recovery phase.

**Table S3.** Peak Value of Different Ion Currents in the Gray Zone (EPI) between TT2 and Tomek models. Epi: epicardial

| **Model** | **INa(pA/pF)** | **I_Kr_(pA/pF)** | **I_Ks_(pA/pF)** | **I_CaL_(pA/pF)** | **[Ca2+]i(μM)** |
| --- | --- | --- | --- | --- | --- |
| TT2 | -166.892 | 0.252 | 0.1818 | -3.660 | 0.1224 |
| Tomek | -135.257 | 0.354 | 0.0042 | -2.987 | 0.1147 |

**APD Restitution in the Gray Zone in epicardial cell**

Figure S5G,H depict APD restitution curves using S1–S2. Tomek’s APD is smaller than TT2’s for all DI values (Figure S5G). The slope of APD in the Tomek model was larger than the TT2 model when DI > 130 ms (Figure S5H). Figure S5I,J) show the dynamic APD restitution curves and slopes, revealing that Tomek’s APD is consistently lower than TT2’s (Figure S5I), and the difference increases with smaller DI. Tomek’s APD slope surpasses TT2’s when DI > 130 ms (Figure S5J).


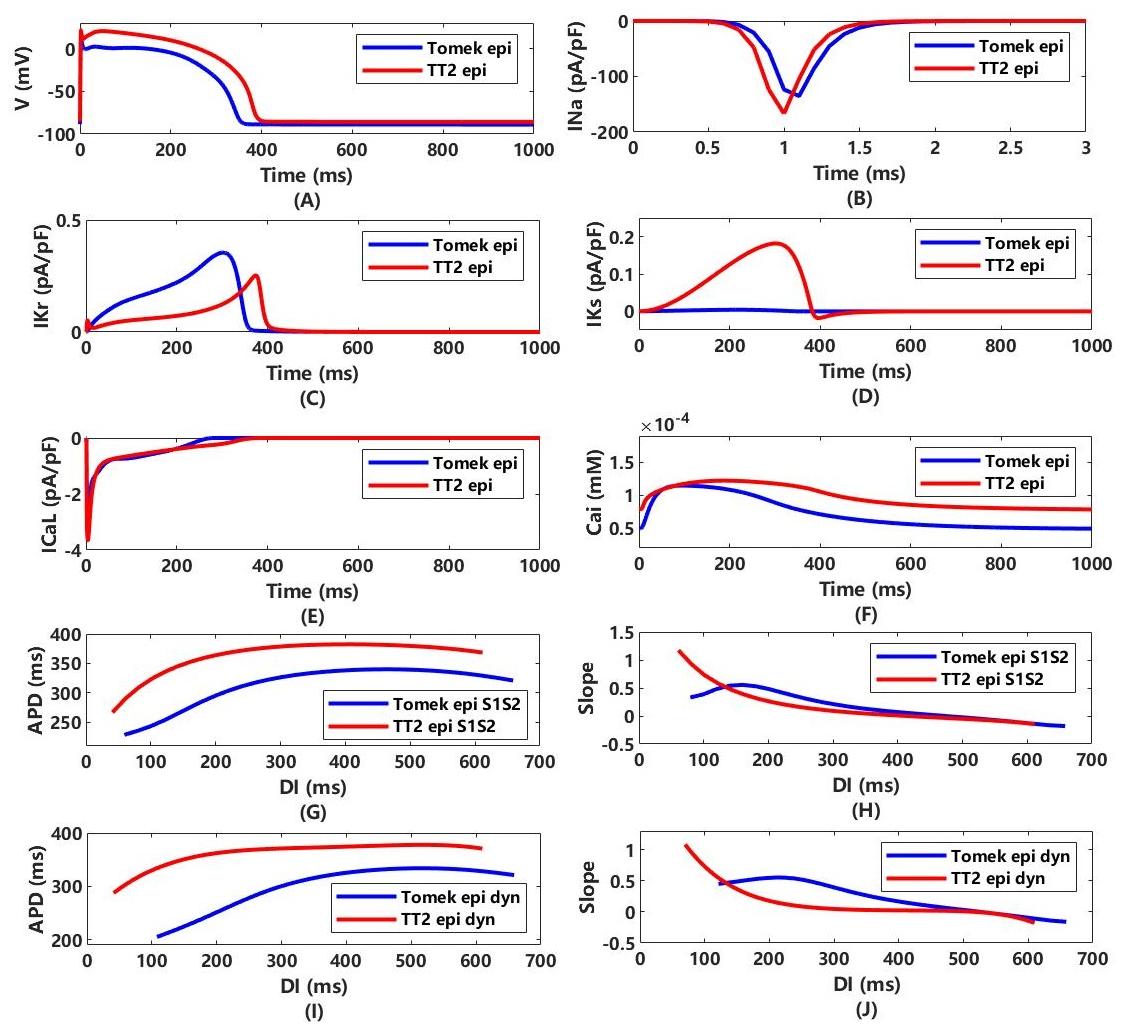


**Figure S5.** Properties of grey zone in the Tomek and the TT2 models (EPI) with 10 µs timestep. (A) membrane potentials, (B-F) major ion currents modified in the model of gray zone, including I_Na_, I_Kr_, I_Ks_, I_K1_, I_CaL_ and [Ca^2+^]_i_, (G) APD restitution curves in S1-S2 protocol, (H) the slope of APD restitution curves in S1-S2 protocol, (I) APD restitution curves in dynamic protocol, (J) the slope of APD restitution curves in dynamic protocol. APD represents APD_90_; DI: diastolic interval; S1S2: S1-S2 protocol; dyn: dynamic protocol; EPI: epicardial.

**Electrophysiological Properties of the Gray Zone in middle cell**

Figure S6 illustrates the action potential and key ion currents for the Tomek and TT2 models in the gray zone of middle cell. The TT2 model has a higher peak voltage (23.34 mV vs. 14.76 mV in Tomek), a longer APD (462.03 ms vs. 455.59 ms), and a less negative resting potential (-85.83 mV vs. -88.85 mV). Table S4 lists the values of various ion currents in the gray zone between the Tomek and TT2 models.

- INa: Smaller in Tomek (-129.388 pA/pF) compared to TT2 (-167.548 pA/pF).
- IKr: TT2 presents a narrow early spike with slightly higher magnitude (0.252 pA/pF vs. 0.240 pA/pF).
- IKs: Significantly higher in TT2 (0.0597 pA/pF vs. 0.0050 pA/pF).
- ICaL: Larger in TT2 (-3.606 pA/pF) compared to Tomek (-4.542 pA/pF), and it inactivates more rapidly.
- [Ca^2+^]_i_: Higher in TT2 (0.1291 μM vs. 0.2046 μM), and remains elevated for a longer portion of the recovery phase.

**Table S4.** Peak Value of Different Ion Currents in the Gray Zone (MCELL) between TT2 and Tomek models. MCELL: middle cell

| **Model** | **INa(pA/pF)** | **I_Kr_(pA/pF)** | **I_Ks_(pA/pF)** | **I_CaL_(pA/pF)** | **[Ca2+]i(μM)** |
| --- | --- | --- | --- | --- | --- |
| TT2 | -167.548 | 0.252 | 0.0597 | -3.606 | 0.1291 |
| Tomek | -129.388 | 0.240 | 0.0050 | -4.542 | 0.2046 |

**APD Restitution in the Gray Zone in middle cell**

Figures S6G,H depict APD restitution curves using S1–S2. Tomek’s APD is smaller than TT2’s for all DI values (Figure S6G). Their slopes are similar when DI > 140 ms (Figure S6H). Figure S6I,J show the dynamic APD restitution curves and slopes, revealing that Tomek’s APD is consistently lower than TT2’s (Figure S6I), and the difference increases with smaller DI. Tomek’s APD slope surpasses TT2’s when DI > 130 ms (Figure S6J).


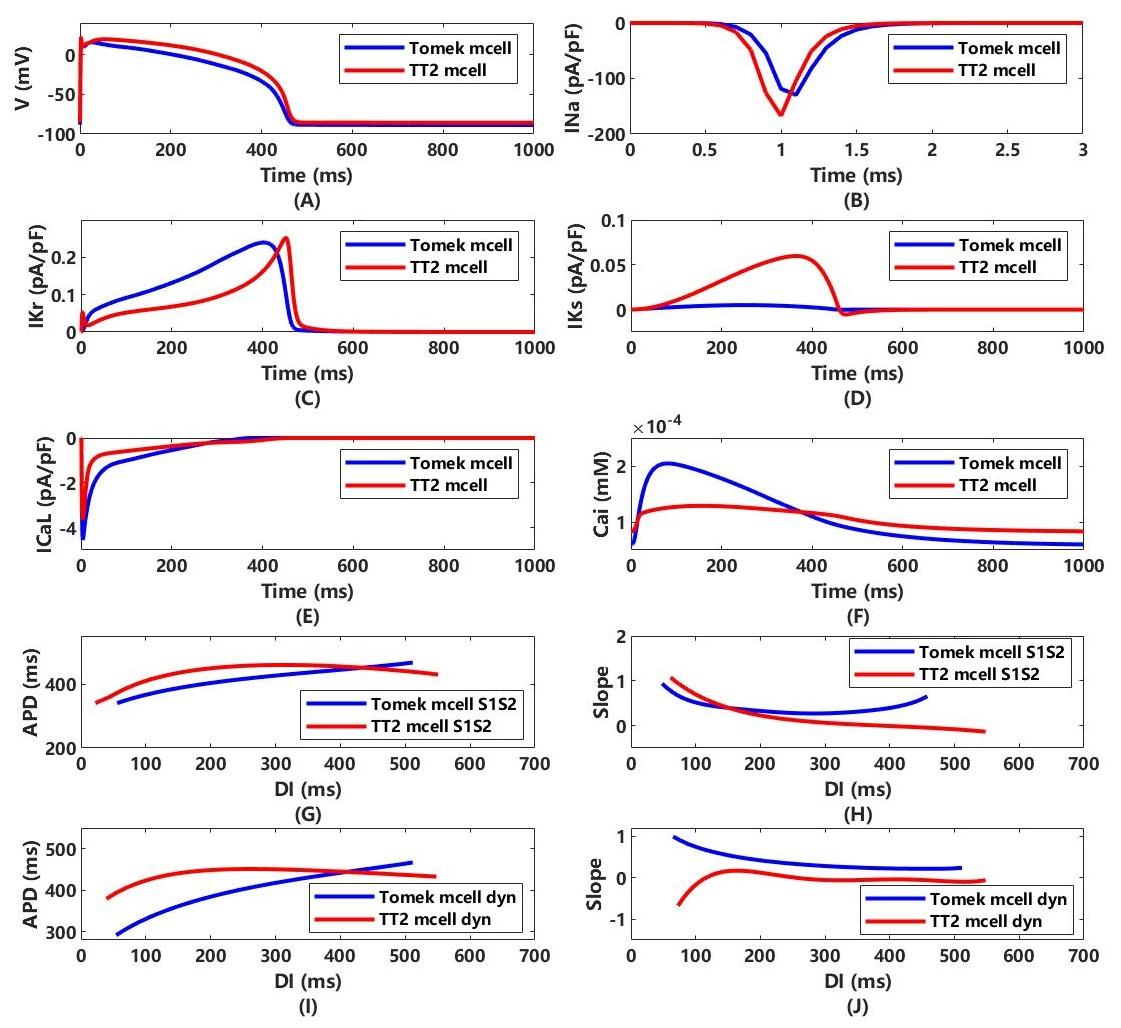


**Figure S6.** Properties of grey zone in the Tomek and the TT2 models (MCELL) with 10 µs timestep. (A) membrane potentials, (B-F) major ion currents modified in the model of gray zone, including I_Na_, I_Kr_, I_Ks_, I_K1_, I_CaL_ and [Ca^2+^]_i_, (G) APD restitution curves in S1-S2 protocol, (H) the slope of APD restitution curves in S1-S2 protocol, (I) APD restitution curves in dynamic protocol, (J) the slope of APD restitution curves in dynamic protocol. APD represents APD_90_; DI: diastolic interval; S1S2: S1-S2 protocol; dyn: dynamic protocol; MCELL: middle cell.

**CV Restitution in Normal and Infarct Tissue of epicardial cell**

Figure S7 compares conduction velocity (CV) in normal and infarct tissue. In normal tissue (Figure S7A, B), the TT2 model shows a faster CV than Tomek for CI > 400 ms. In infarct tissue (Figure S7C, D), the TT2 maintains faster CV and steeper slopes, but both models converge when CI > 700 ms.
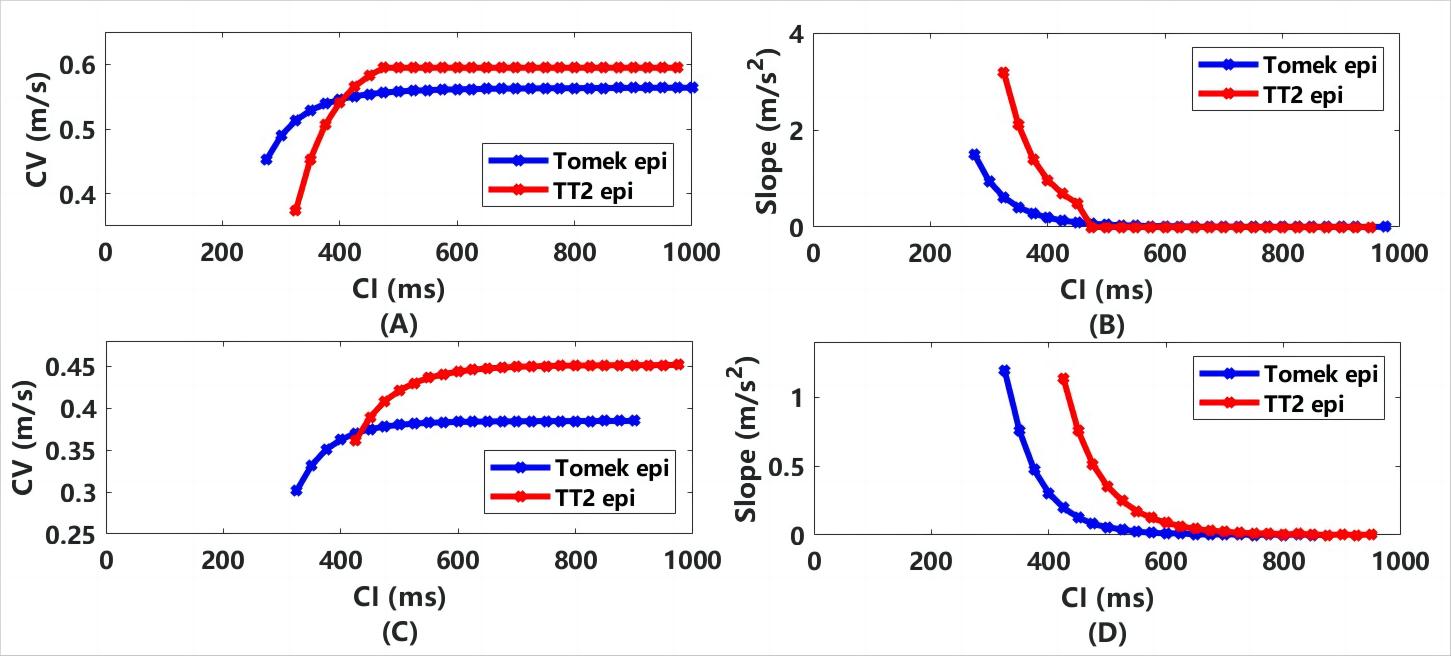


**Figure S7.** CV restitution curves and its slope of normal and infarct tissue in the Tomek and the TT2 models (EPI) measured in a 1D cable with 10 µs timestep. (A) CV restitution curve of normal tissue; (B) the slope of CV restitution curve in normal tissue. (C) CV restitution curve of infarct tissue. (D) the slope of CV restitution curves in infarct tissue. CV: conduction velocity; CI: coupling interval; EPI: epicardial.

**CV Restitution in Normal and Infarct Tissue of middle cell**

Figure S8 compares conduction velocity (CV) in normal and infarct tissue. In normal tissue (Figure S8A, B), the TT2 model shows a faster CV than Tomek for CI > 450 ms. In infarct tissue (Figure S8C, D), the TT2 maintains faster CV and steeper slopes, but both models converge when CI > 750 ms.


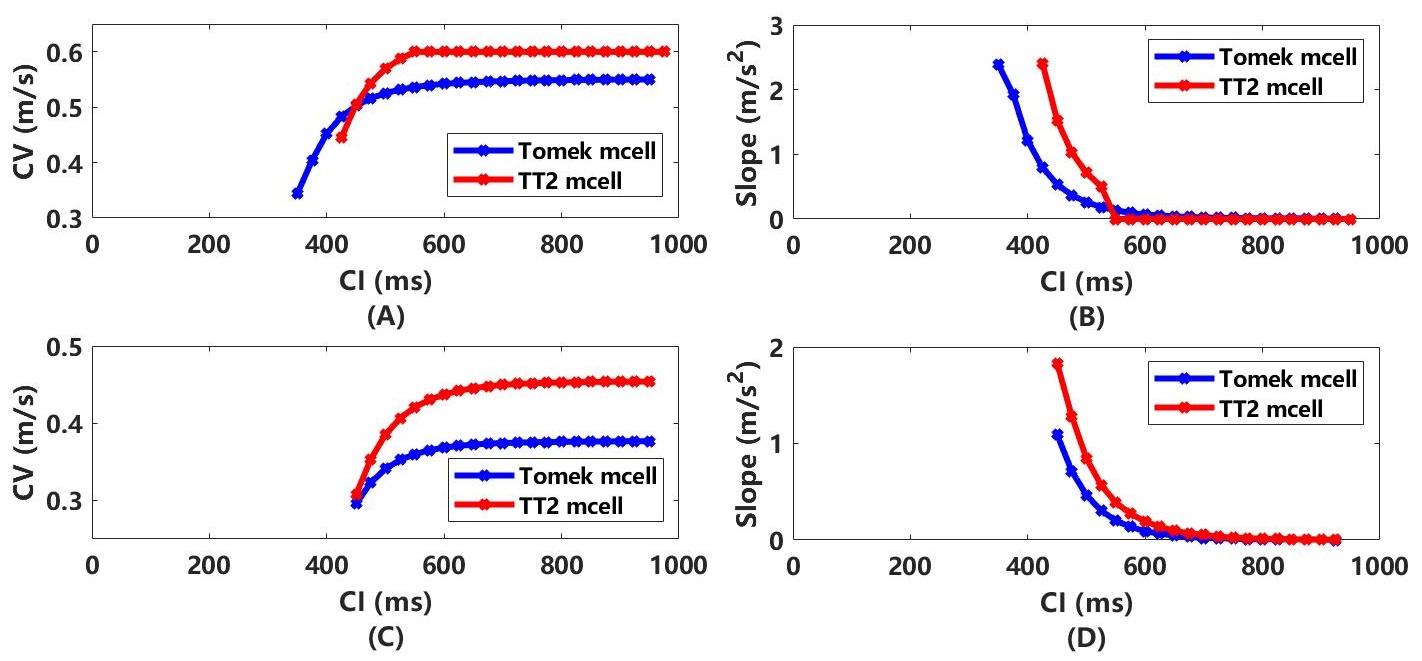


**Figure S8.** CV restitution curves and its slope of normal and infarct tissue in the Tomek and the TT2 models (MCELL) measured in a 1D cable with 10 µs timestep. (A) CV restitution curve of normal tissue; (B) the slope of CV restitution curve in normal tissue. (C) CV restitution curve of infarct tissue. (D) the slope of CV restitution curves in infarct tissue. CV: conduction velocity; CI: coupling interval; MCELL: middle cell.

Video S1. Reentry induced in the 2D simulation with Tomek endocardial normal cell model and timestep of 5, 10, 15, 20, 25 and 30 µs.

Video S2. Reentry induced in the 2D simulation with TT2 endocardial normal cell model and timestep of 5, 10, 15, 20, 25 and 30 µs.

Video S3. [Ca^2+^]_i_ induced in the 2D simulation with TT2 endocardial normal cell model and timestep of 10 µs.

Video S4. [Ca^2+^]_i_ induced in the 2D simulation with Tomek endocardial normal cell model and timestep of 10 µs.

Video S5. Reentry induced in the 2D simulation with TT2 endocardial gray zone cell model and timestep of 10 µs.

Video S6. Reentry induced in the 2D simulation with Tomek endocardial gray zone cell model and timestep of 10 µs.

Video S7. Reentry 1 induced in the 3D simulation with TT2 endocardial normal and gray zone cell model. The reentry locates at the posterior wall of left ventricle.

Video S8. Reentry 2 induced in the 3D simulation with TT2 endocardial normal and gray zone cell model. The reentry locates at the anterior lateral wall of left ventricle.

Video S9. Reentry 3 induced in the 3D simulation with TT2 endocardial normal and gray zone cell model. The reentry locates in the upper front wall of left ventricle.

Video S10. Reentry 1 induced in the 3D simulation with Tomek endocardial normal and gray zone cell model. The reentry locates at the posterior wall of left ventricle.

Video S11. Reentry 2 induced in the 3D simulation with Tomek endocardial normal and gray zone cell model. The reentry locates at the anterior lateral wall of left ventricle.

Video S12. Reentry 3 induced in the 3D simulation with Tomek endocardial normal and gray zone cell model. The reentry locates in the lower part of the anterior wall of left ventricle.
